# Supplementary material for: Efficient CRISPR-Cas9 based cytosine base editors for phytopathogenic bacteria
Source: Commun Biol. 2023 Jan 17;6:56. doi: 10.1038/s42003-023-04451-8 (PMC9842757; doi:10.1038/s42003-023-04451-8)
Supplement: Supplementary file 2 — Supplementary Information [file 42003_2023_4451_MOESM2_ESM.pdf]

Supplementary Information for

Efficient CRISPR-Cas9 based cytosine base editors for phytopathogenic bacteria

Chenhao Li[1,2,7], Longfei Wang[1,7], Leland J. Cseke[1], Fernanda Vasconcelos[1], Jose Carlos Huguet-Tapia[3], Walter Gassmann[1], Laurens Pauwels[4,5], Frank White[3], Hansong Dong[2], Bing Yang[1,6,*]

[1] Division of Plant Science and Technology, Bond Life Sciences Center, University of Missouri, Columbia, Missouri, USA

[2] Department of Plant Pathology, Nanjing Agricultural University, Nanjing, Jiangsu, P.R. China

[3] Department of Plant Pathology, University of Florida, Gainesville, Florida, USA

[4] Department of Plant Biotechnology and Bioinformatics, Ghent University, B-9052 Ghent, Belgium

[5] Center for Plant Systems Biology, VIB, B-9052 Ghent, Belgium

[6] Donald Danforth Plant Science Center, St. Louis, Missouri, USA

[7]These authors contributed equally.

***Email:** yangbi@missouri.edu

This PDF file includes:

Supplementary text
Supplementary Figures 1 to 26
Supplementary Tables 1 to 3
Supplementary References

Supporting Figures

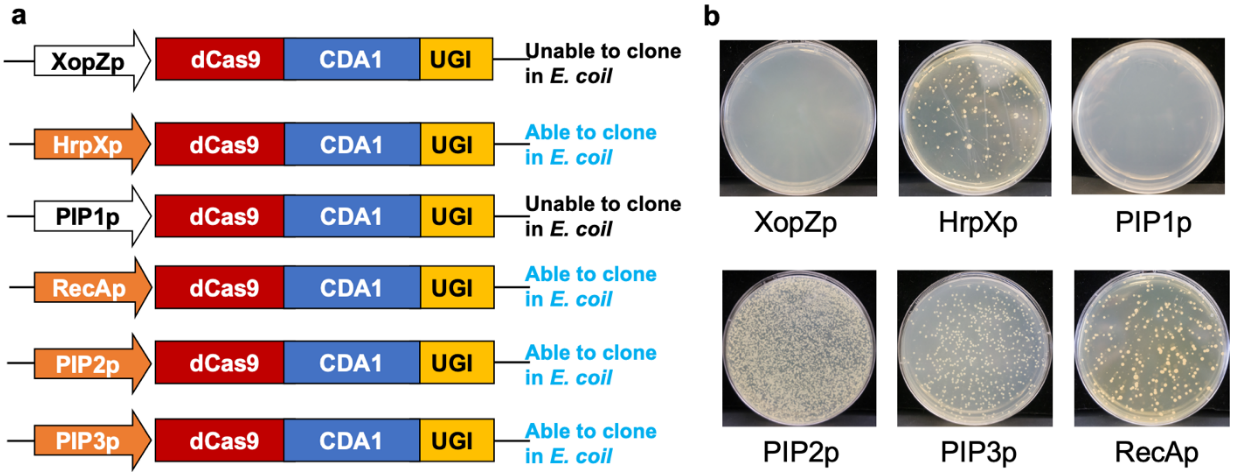

Supplementary Figure 1. Identification of promoters for base editors in *Xanthomonas*.

a Six different promoters were selected from *X. oryzae* pv. *oryzae* PXO99^{A} for expression of dCas9-CDA1-UGI; XopZp from the type III effector gene *xopZ*; HrpXp from *hrpX* gene; PIP1p, PIP2p, and PIP3p from plant inducible gene 1, 2 and 3, respectively; dCas9, an enzymatically dead Cas9 (D10A, H840A); CDA1, *Petromyzon marinus* cytidine deaminase; and UGI, uracil DNA glycosylase inhibitor.
b Culture plates with successful and failed transformations of *E. coli* with different plasmids as indicated in (*A*). Images were taken 15 hours after bacterial transformation.

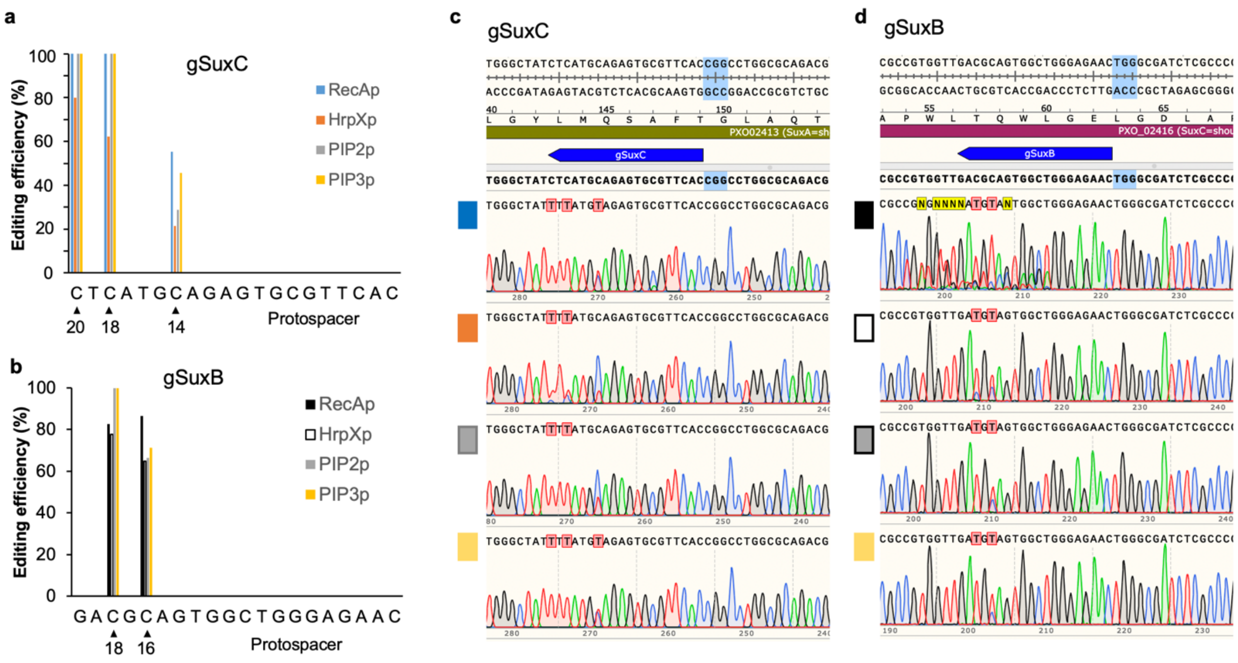

Supplementary Figure 2. Efficient base editing in *Xanthomonas* by base editors driven by four promoters.

a, b Quantification of the C to T editing before curing for gSuxC and gSuxB in PXO99^{A}, respectively. dCas9-CDA-UGI was driven by four promoters RecAp, HrpXp, PIP2p and PIP3p, individually. The percentage of C to T conversion is based on the Sanger sequencing chromatograms. Edited C's in the protospacers are indicated by solid triangles with the numbers for the positions relative to the PAM.

c, d Sequencing chromatograms (partial sections) of PCR amplicons from the target regions of *suxC* and *suxB*, respectively. The promoters for dCas9-CDA-UGI are indicated at the left sides of chromatograms. Three independent transformants were pooled for genomic DNA extraction and PCR-amplification for each construct.

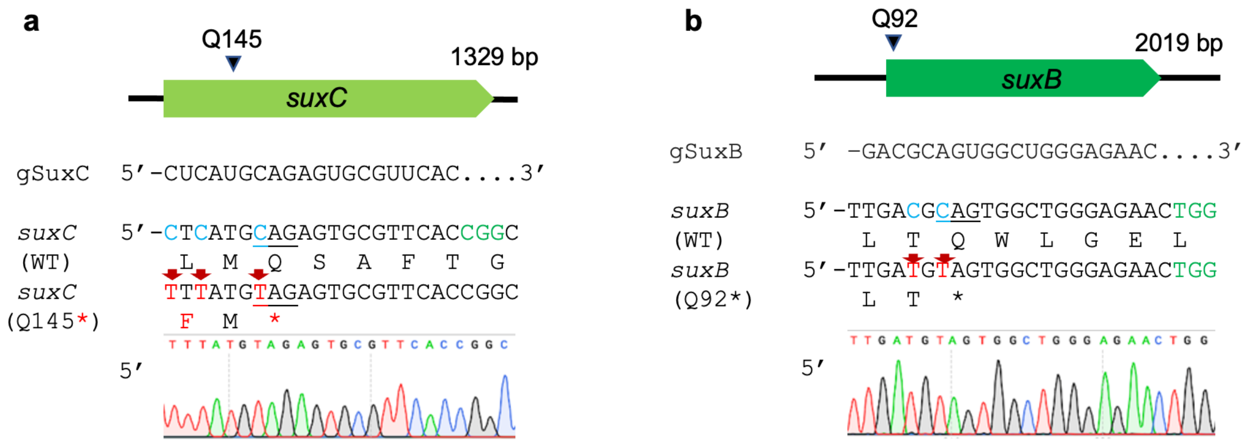

Supplementary Figure 3. Editing outcomes of *suxC* and *suxB* in PXO99^{A} induced by dCas9-CDA1-UGI.

a, b Base editing outcomes after eviction of editors; codons for glutamine (Q) in *suxC* and *suxB* are underlined. Guide RNAs and target sequences with PAM (in green) are also shown. Converted T (in red) from C (in blue) is indicated. Single letters for amino acids are used with asterisk for stop codon. Examples of sequencing chromatograms for *suxC* and *suxB* are provided to show the C to T conversion.

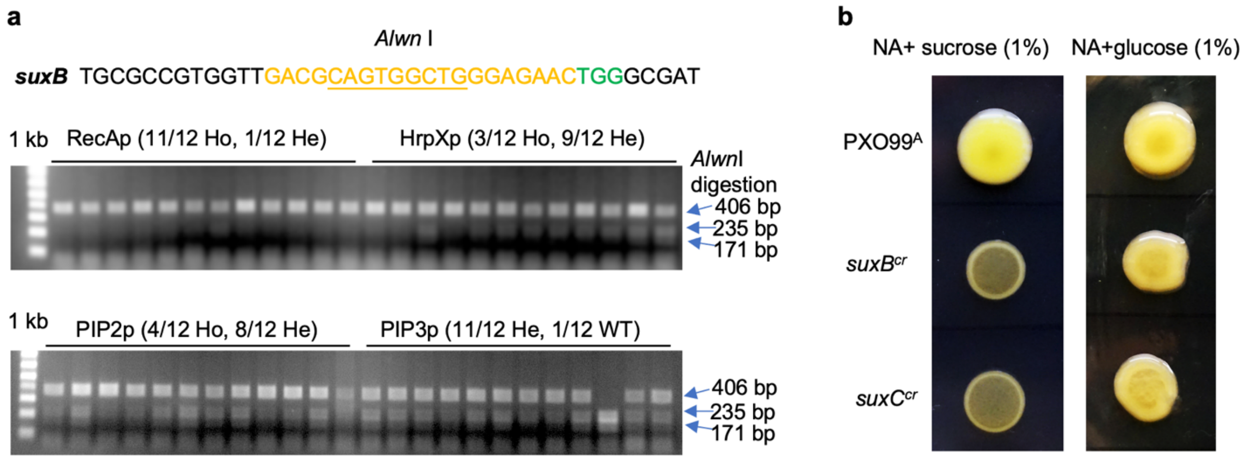

Supplementary Figure 4. Editing efficiency of *suxB* in PXO99^{A} induced by dCas9-CDA1-UGI under four different promoters.

a Base editing of *suxB* was assessed by restriction of PCR-amplicons. gSuxB-targeted region of *suxB* is shown with PAM (green), protospacer (orange) and *Alwn*I recognition sequence (underlined). The genomic region of *suxB* was PCR-amplified and digested with *Alwn*I. Wild-type PCR product (406 bp) was be digested completely to form two smaller bands (235 bp and 171 bp), and the edited PCR-amplicons were either fully or partially cut by *Alwn* I. Ho: Homogenous edits; He: Heterogenous edits.
b Colony morphology of PXO99^{A} and its *sux* representative *suxB* and *suxC* mutants on culture plates supplemented with nutrient broth and 1% sucrose (left panel) or 1% glucose (right panel).

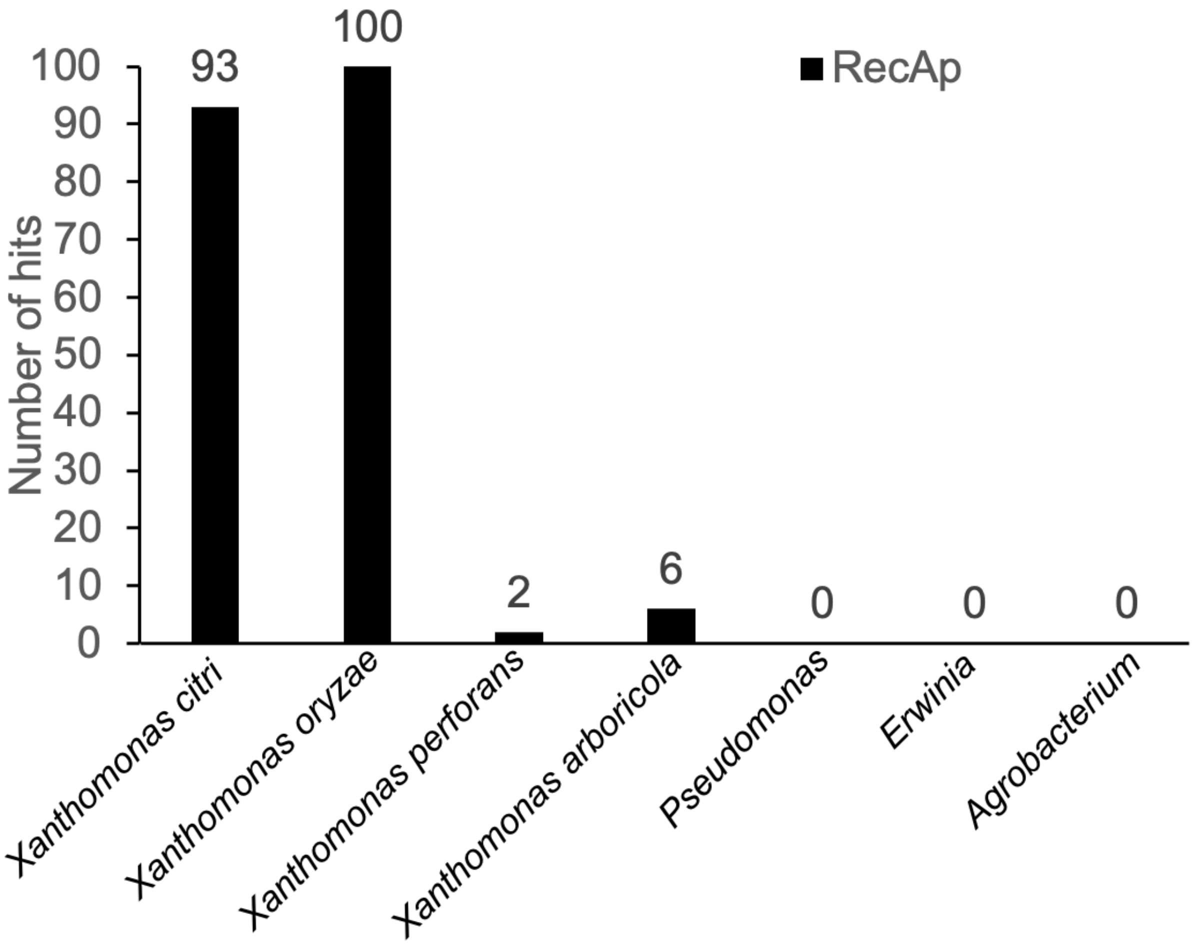

Supplementary Figure 5. *recA* promoter sequences in different bacterial species.

The numbers of hits represent the number of isolates in bacterial species containing the sequences homologous to *recA* promoter, the data is from NCBI (National Center for Biotechnology Information).

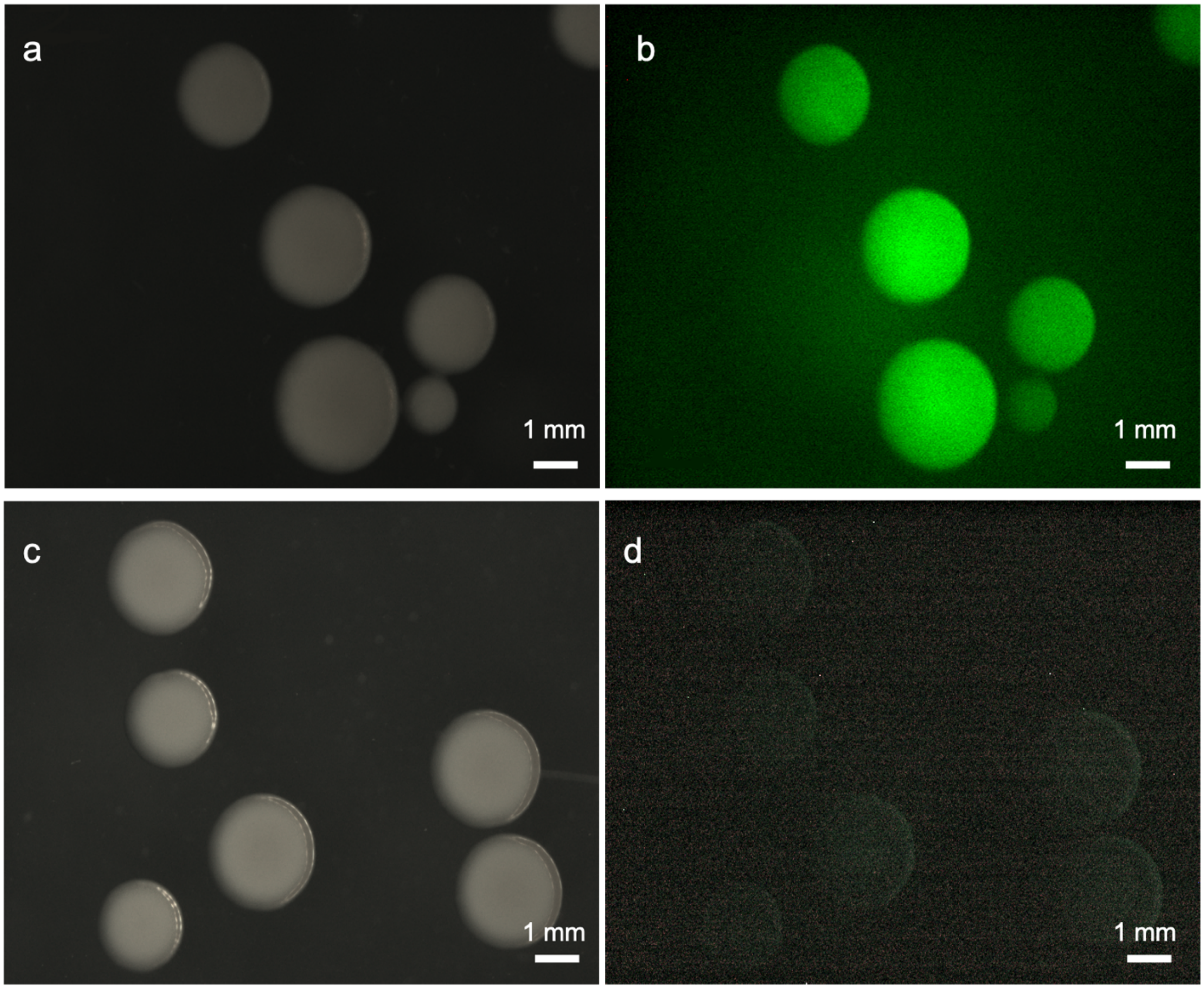

Supplementary Figure 6. GFP fluorescence facilitates identification of transformants and eviction of plasmid.

a, b Colonies of *Agrobacterium* LBA4404 transformed with GFP-tagged CBE. Images were taken under bright field (**a**) and green fluorescence light (**b**). Colonies were analyzed 3 days of transformation.
c, d Colonies from plasmid evicted LBA4404 cells under bright field (**c**) and green fluorescence light (**d**). To evict the plasmid, bacterial cells were grown with LB liquid medium containing 10% sucrose for 2 days, then plated on LB medium containing 10% sucrose for single colonies. Scale bars denote 1 mm.

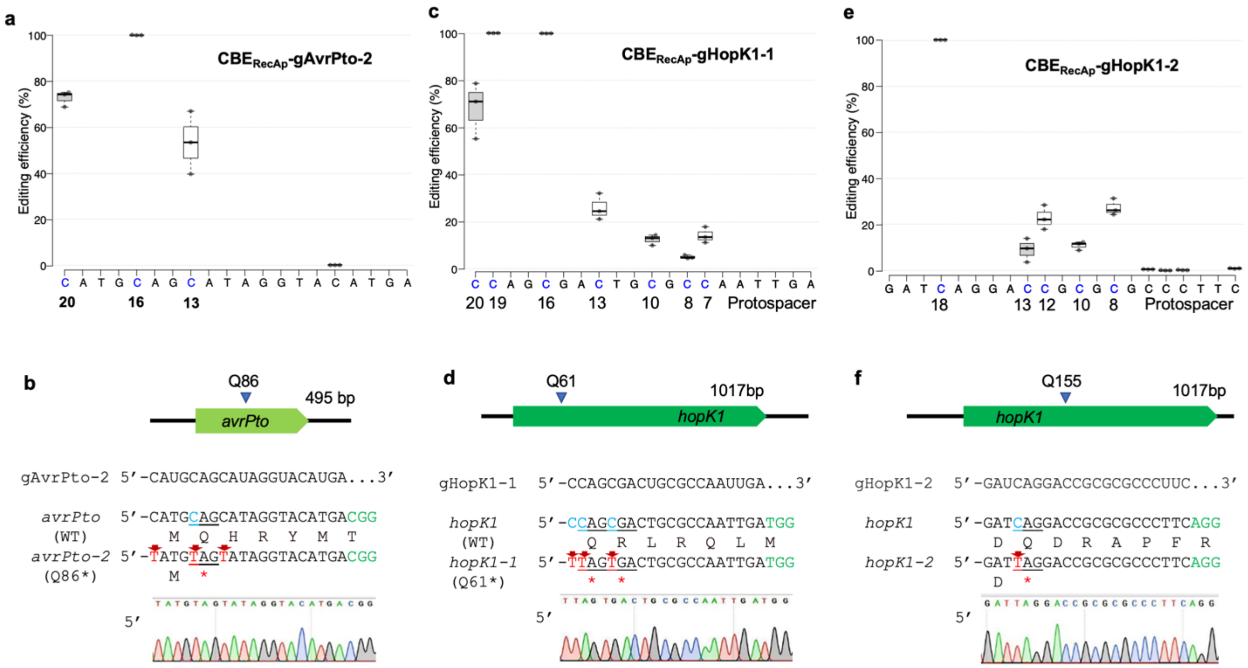

Supplementary Figure 7. Base editing of *avrPto* and *hopK* in *Pst* DC3000.

a, c, e Quantification of the C to T editing before eviction of gAvrPto-2, gHopK1-1 and gHopK1-2, respectively. The percentage of C to T conversion is based on deep sequencing of 15 individual transformants in three pools. Edited C's in the protospacers are indicated by with the numbers for the positions relative to the PAM.

b, d, f Base editing outcomes after eviction of editor. Guide RNAs and target sequences with PAM (in green) are also shown. Converted T (in red) from C (in blue) is indicated. Single letters for amino acids are used with asterisk for stop codon. An example of a chromatogram for each target site is provided to show the C to T conversion for each of guide RNA.

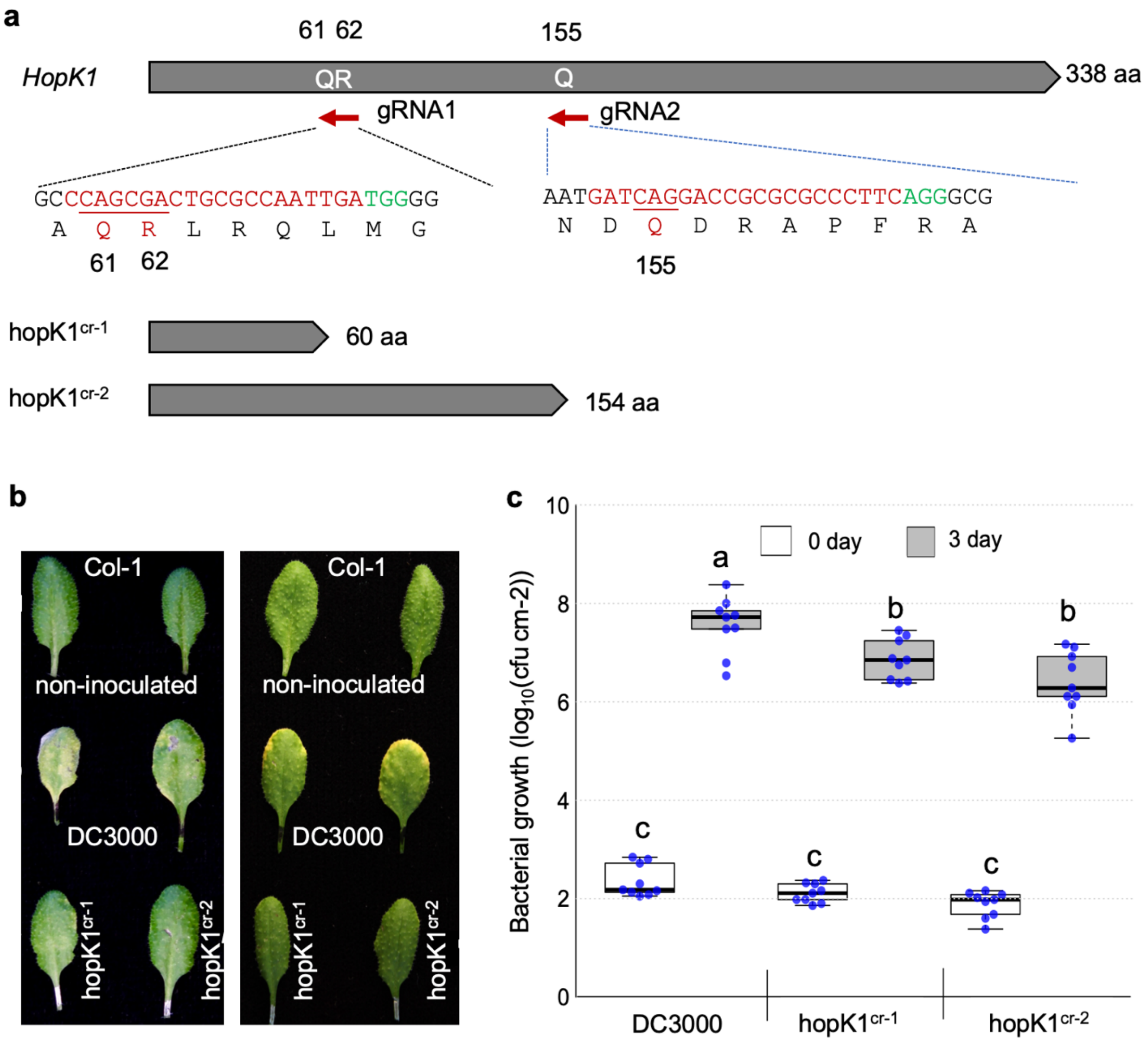

Supplementary Figure 8. Phenotypes of *hopK1* mutants in Arabidopsis Col-1

a Schematic of *hopK1* and the CBE-induced deletions resulting in $hopK1^{cr\text{-}1}$ and $hopK1^{cr\text{-}2}$.
b Replicated disease responses of Arabidopsis accession Col-1 when infiltrated at a bacterial density of $1x10^6$ cfu/ml with DC3000 compared to decreased responses observed using $hopK1^{cr\text{-}1}$ and $hopK1^{cr\text{-}2}$.
c *In planta* bacterial growth assay in Col-1 infiltrated with DC3000, $hopK1^{cr\text{-}1}$ or $hopK1^{cr\text{-}2}$ at a bacterial density of $5x10^4$ cfu/ml. Boxes extend from 25th to 75th percentiles and display median values as center lines on the logarithmic scale. Whisker plots show the minimum and maximum values along with individual data points. Three independent experiments each with triplicate samples were performed, and treatments with different lowercases are significantly different at $p<0.05$.

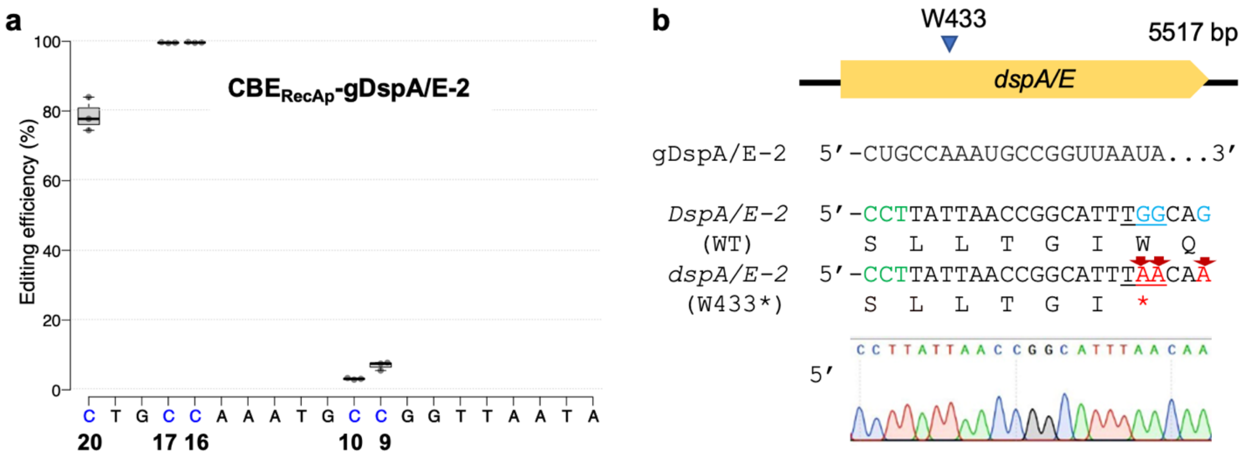

Supplementary Figure 9. Efficient base editing in *Erwinia*.

a Quantification of the C to T editing before eviction of gDspA/E-2. The percentage of C to T conversion is based on deep sequencing of 15 individual transformants in three pools. Edited C's in the protospacers are indicated by with the numbers for the positions relative to the PAM.

b Base editing outcomes after eviction of editor. Guide RNAs and target sequences with PAM (in green) are also shown. Converted T (in red) from C (in blue) is indicated. Single letters for amino acids are used with asterisk for stop codon. An example of a chromatogram for each target site is provided to show the C to T conversion for each of guide RNA. Please note that the protospacer sequence in (**a**) is the flip view of target sequences in (**b**).

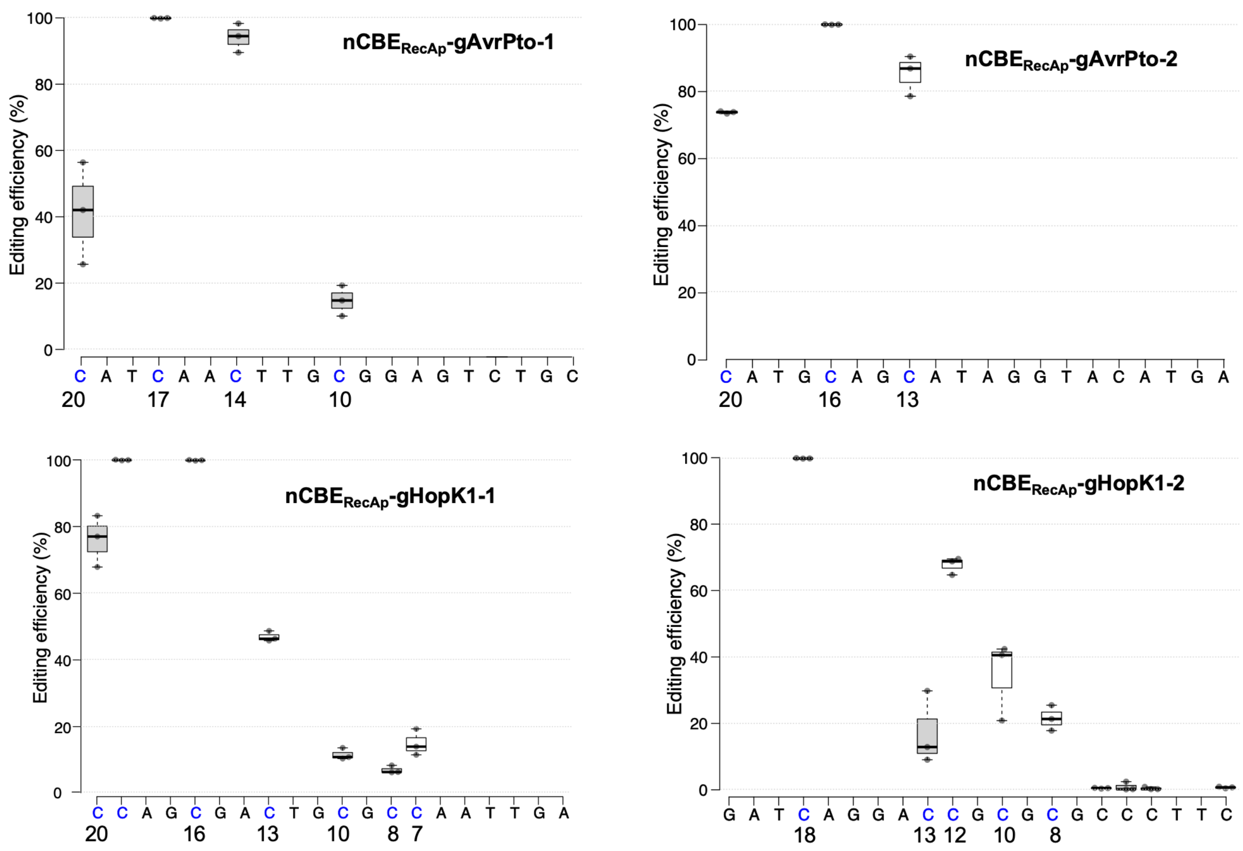

Supplementary Figure 10. Efficient base editing in *Pseudomonas* by Cas9 nickase based editor.

Quantification of the C to T editing before eviction of gRNA as indicated. The percentage of C to T conversion is based on deep sequencing of 15 individual transformants in three pools. Edited C's in the protospacers are indicated by with the numbers for the positions relative to the PAM.

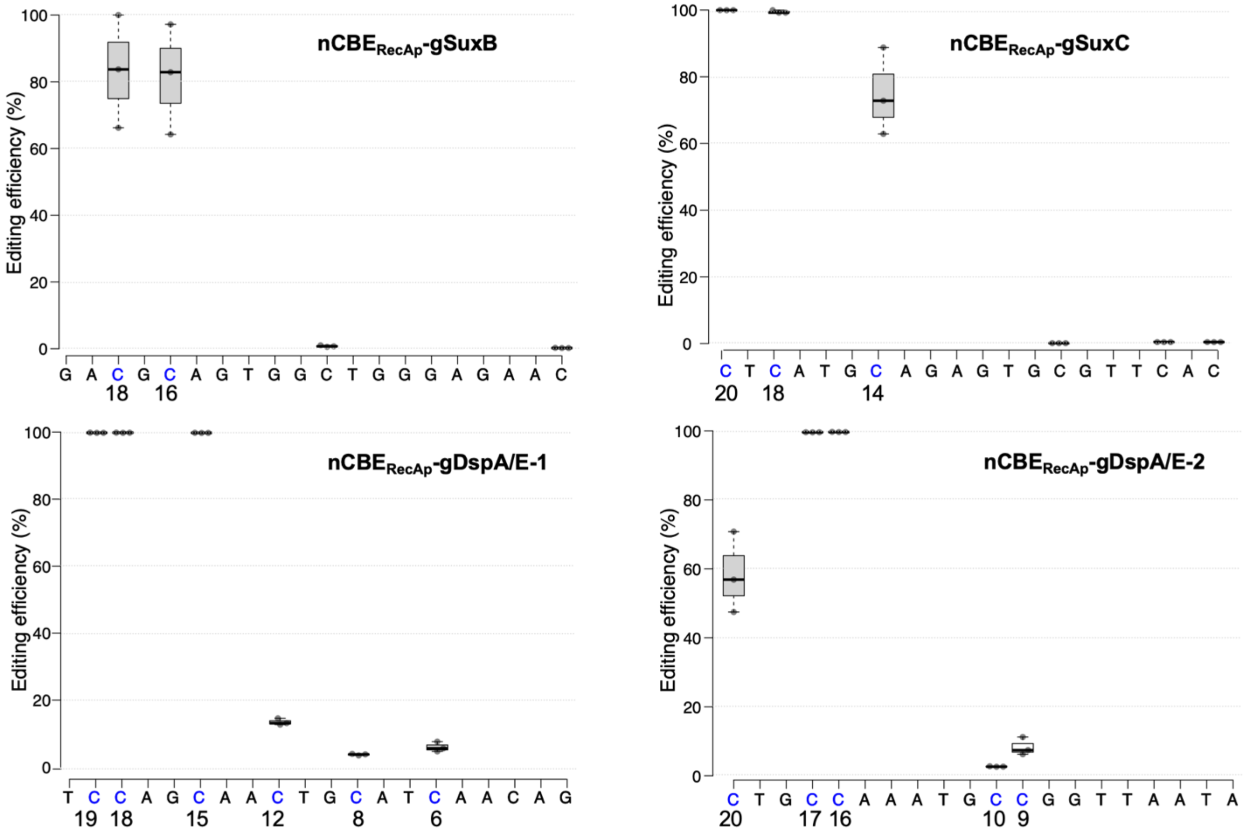

Supplementary Figure 11. Efficient base editing in *Xanthomonas* and *Erwinia* by Cas9 nickase based editor.
Quantification of the C to T editing before eviction of gRNA as indicated. The percentage of C to T conversion is based on deep sequencing of 15 individual transformants in three pools. Edited C's in the protospacers are indicated by with the numbers for the positions relative to the PAM.

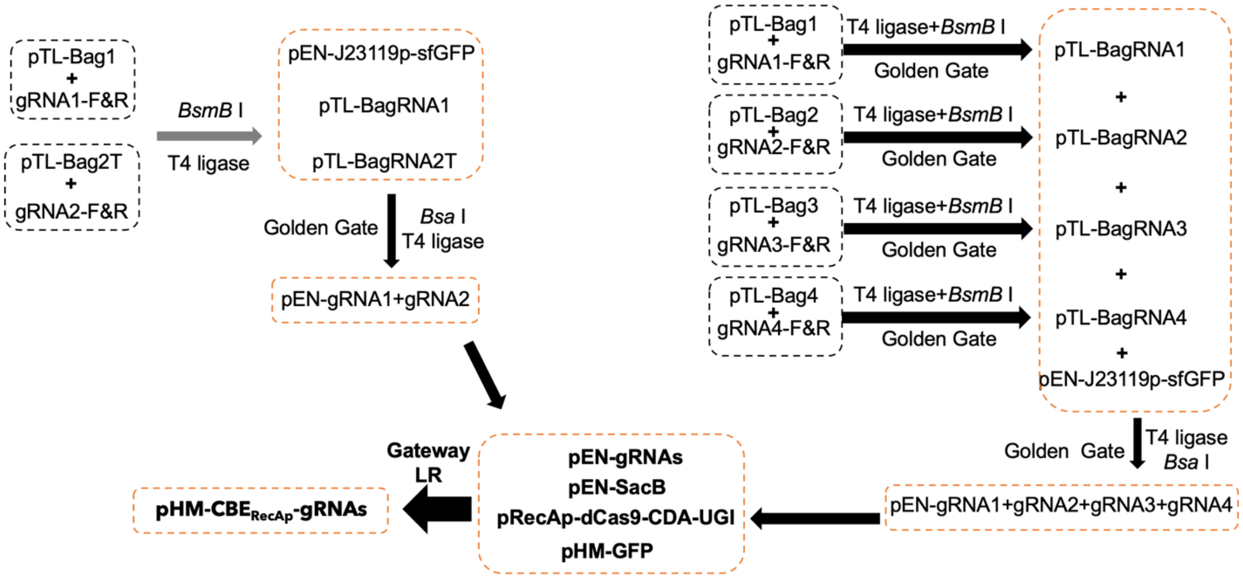

Supplementary Figure 12. Flowchart to construct multiplex base editors.

Base editor with two or four guide RNAs can be made from two or four oligo-derived double-stranded fragments and corresponding two or four gRNA modular units (left panel, right panel), via *BsmB*I restriction and T4 ligation, resulting in pEN-gRNA1+gRNA2 or pEN-gRNA1+gRNA2+gRNA3+gRNA4, respectively. The pEN-gRNAs can be further combined with other three components (pEN-SacB, pRecAp-dCas9-CDA-UGI, and pHM-GFP) into single base editor.

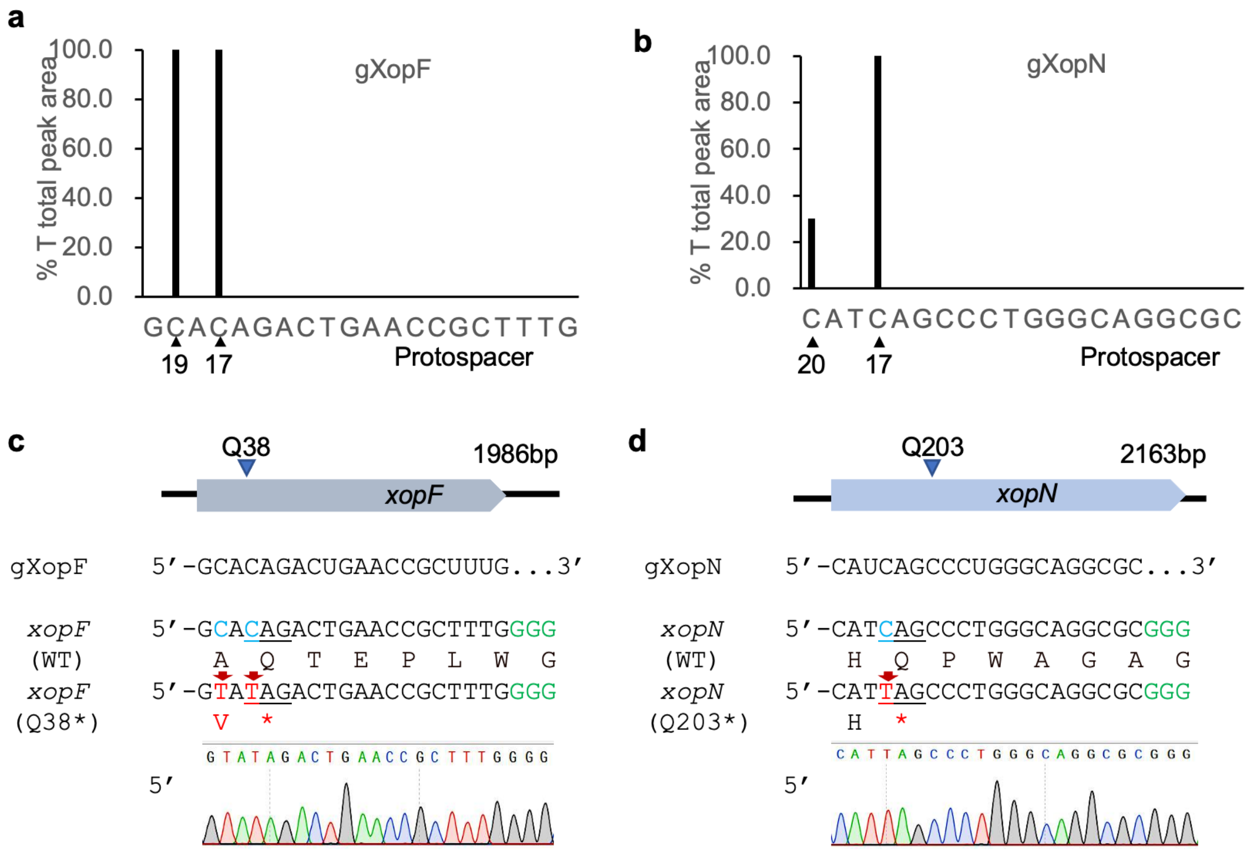

Supplementary Figure 13. Two-gRNA-based editing of *xopF* and *xopN* in PXO99^{A}.
a, b Quantification of the C to T editing before curing for gXopF and gXopN, respectively. The percentage of C to T conversion is based on the Sanger sequencing chromatographs for gXopF (n=8) and gXopN (n=8). Edited C's in the protospacers are indicated by solid triangles with the numbers for the positions relative to the PAM.
c, d Base editing outcomes after eviction of editor. Guide RNAs and target sequences with PAM (in green) are also shown. Converted T (in red) from C (in blue) is indicated. Single letters for amino acids are used with asterisk for stop codon. An example of a chromatogram for *xopF* and *xopN* is provided to show the C to T conversion for each of guide RNA.

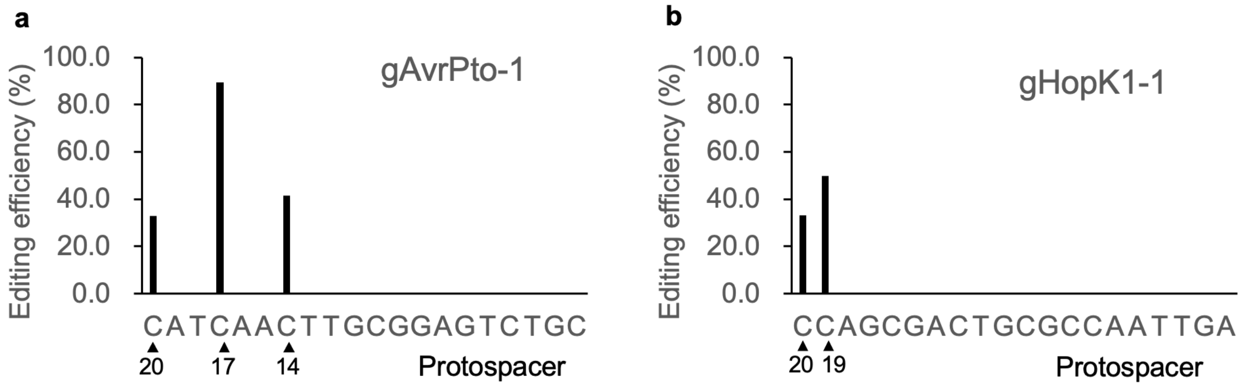

Supplementary Figure 14. Two-gRNA-based editing of *avrPto* and *hopK* in *Pst* DC3000.

Quantification of the C to T editing before curing for gAvrPto-1 (**a**) and gHopK1-1 (**b**), respectively. The percentage of C to T conversion is based on the Sanger sequencing chromatographs for gAvrPto-1 (n=6) and gHopK1-1 (n=6). Edited C's in the protospacers are indicated by solid triangles with the numbers for the positions relative to the PAM.

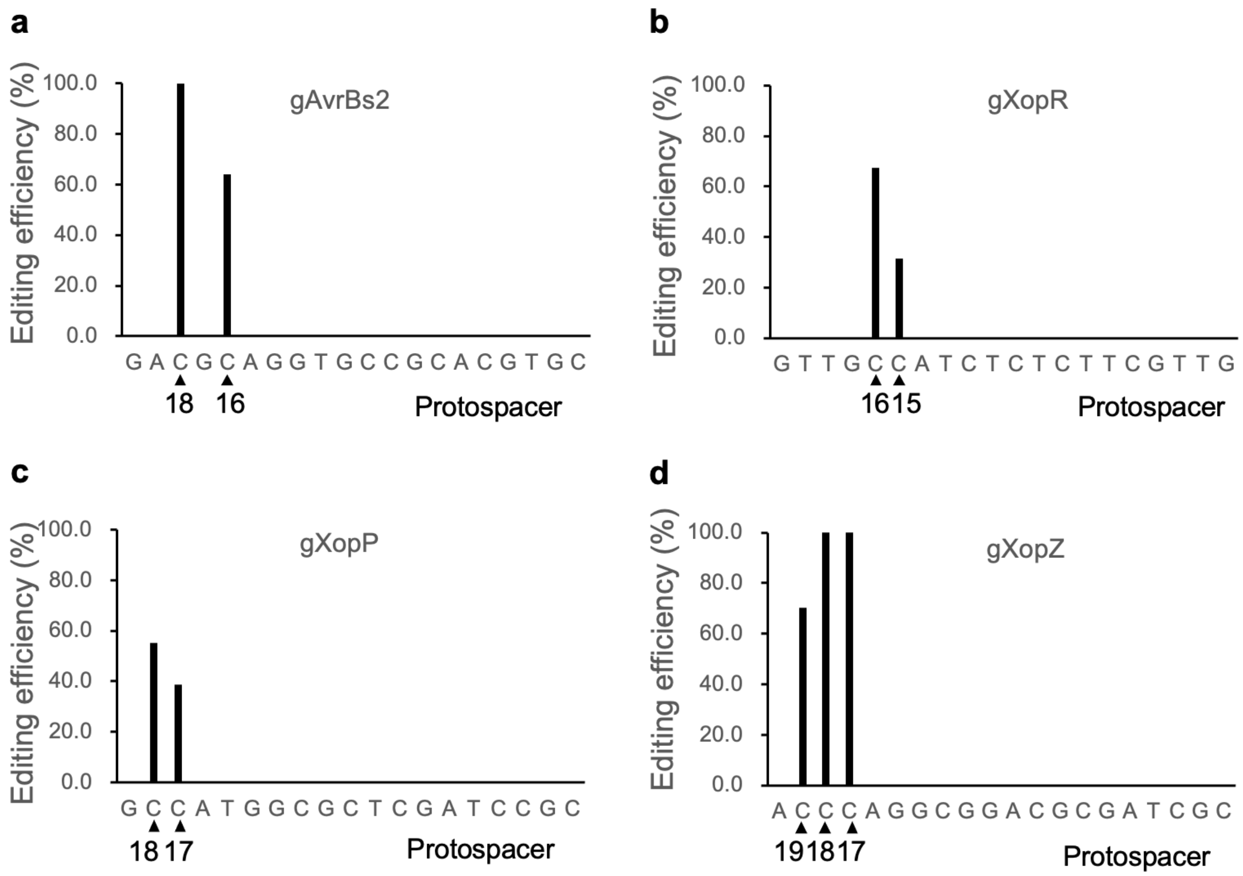

Supplementary Figure 15. Four-gRNA-based editing of *avrBs2*, *xopR*, *xopP* and *xopZ* in PXO99^{A}.

Quantification of the C to T editing before eviction of gAvrBs2 (**a**), gXopR (**b**), gXopP (**c**) and gXopZ (**d**), respectively. The percentage of C to T conversion is based on the Sanger sequencing chromatographs for individual gRNAs in different colonies (n=3). Edited C's in the protospacers are indicated by solid triangles with the numbers for the positions relative to the PAM.

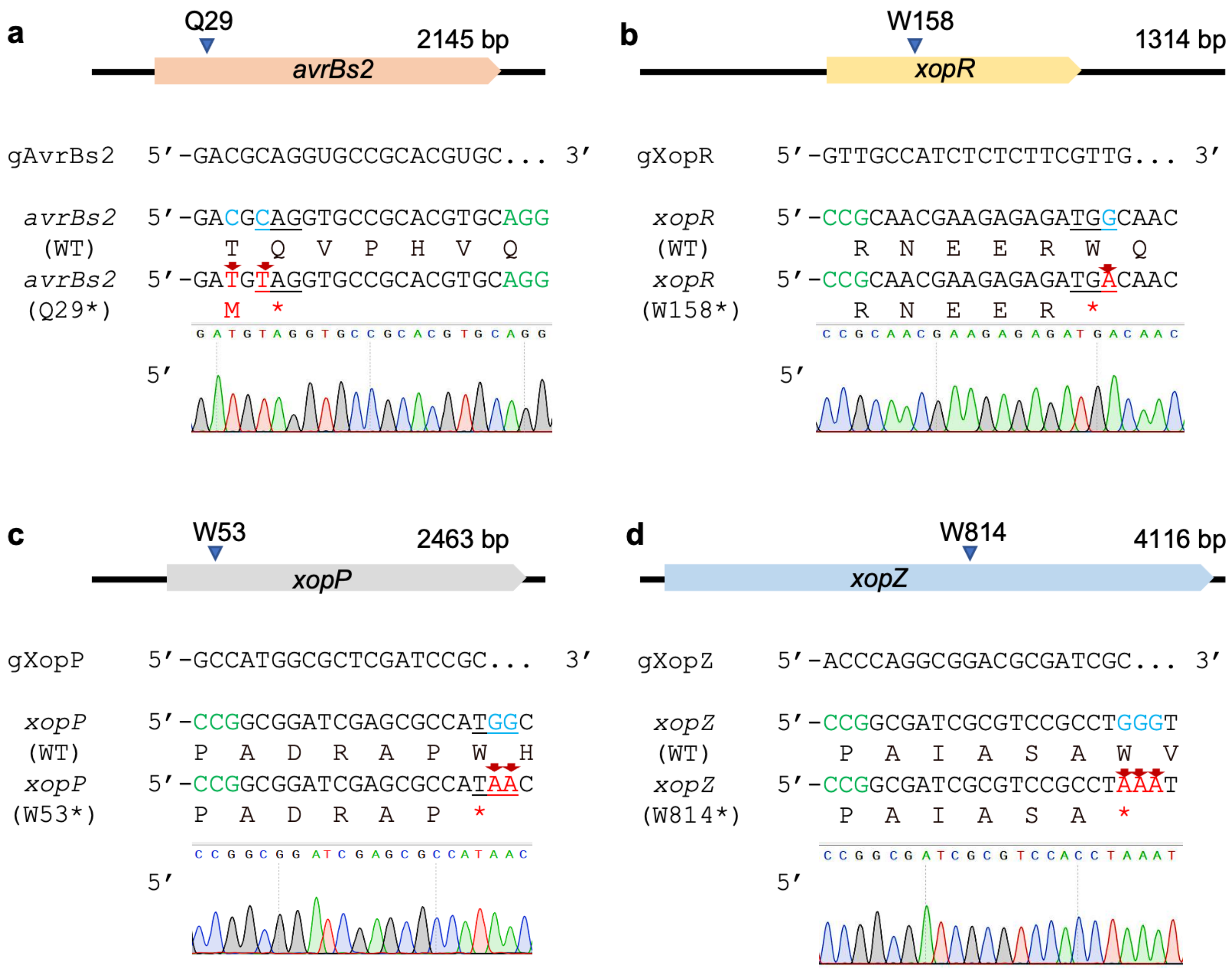

Supplementary Figure 16. Genotypes of based edited *avrBs2*, *xopR*, *xopP* and *xopZ* in PXO99^{A}.

Outcomes of the C to T editing after eviction of guide RNAs, gAvrBs2 (**a**), gXopR (**b**), gXopP (**c**) and gXopZ (**d**). Guide RNAs and target sequences with PAM (in green) are also shown. Converted T (in red) from C (in blue) is indicated. Single letters for amino acids are used with asterisk for stop codon. An example of a chromatogram for each type III effector gene is provided to show the C to T conversion for each of guide RNA.

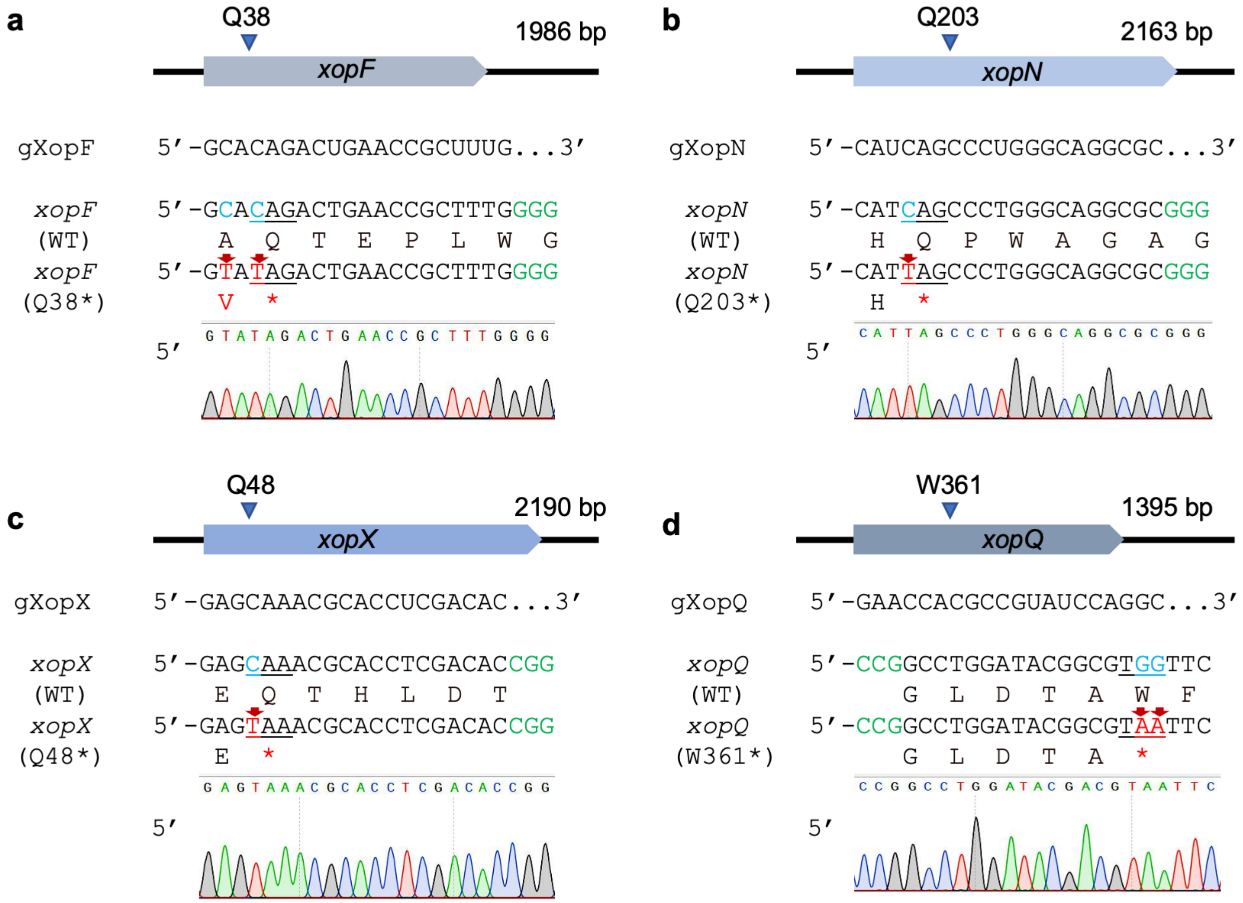

Supplementary Figure 17. Genotypes of based edited *xopF*, *xopN*, *xopX* and *xopQ* in quadruple mutant of *avrBs2/xopR/xopP/xopZ*.

Outcomes of the C to T editing after eviction of guide RNAs, gXopF (**a**), gXopN (**b**), gXopX (**c**) and gXopQ (**d**). Guide RNAs and target sequences with PAM (in green) are also shown. Converted T (in red) from C (in blue) is indicated. Single letters for amino acids are used with asterisk for stop codon. An example of a chromatogram for each type III effector gene to is provided show the C to T conversion for each of guide RNA.

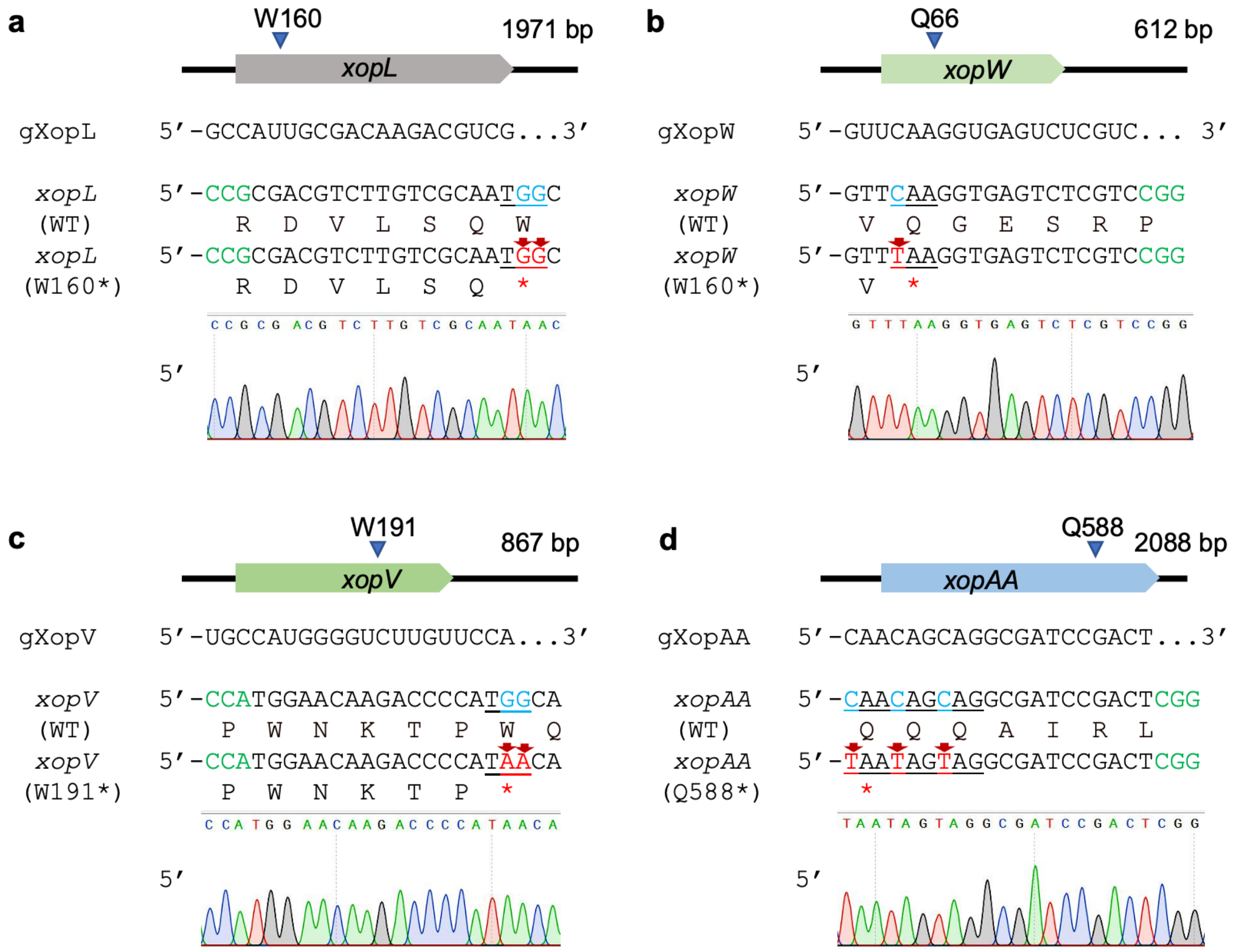

Supplementary Figure 18. Genotypes of based edited *xopL*, *xopW*, *xopV* and *xopAA* in octuple mutant of *avrBs2/xopR/xopP/xopZ/xopF/xopN/xopX/xopQ*.

Outcomes of the C to T editing after eviction of guide RNAs, gXopL (**a**), gXopW (**b**), gXopV (**c**) and gXopAA (**d**). Guide RNAs and target sequences with PAM (in green) are also shown. Converted T (in red) from C (in blue) is indicated. Single letters for amino acids are used with asterisk for stop codon. An example of the chromatogram for each type III effector gene is provided to show the C to T conversion for each of guide RNA.

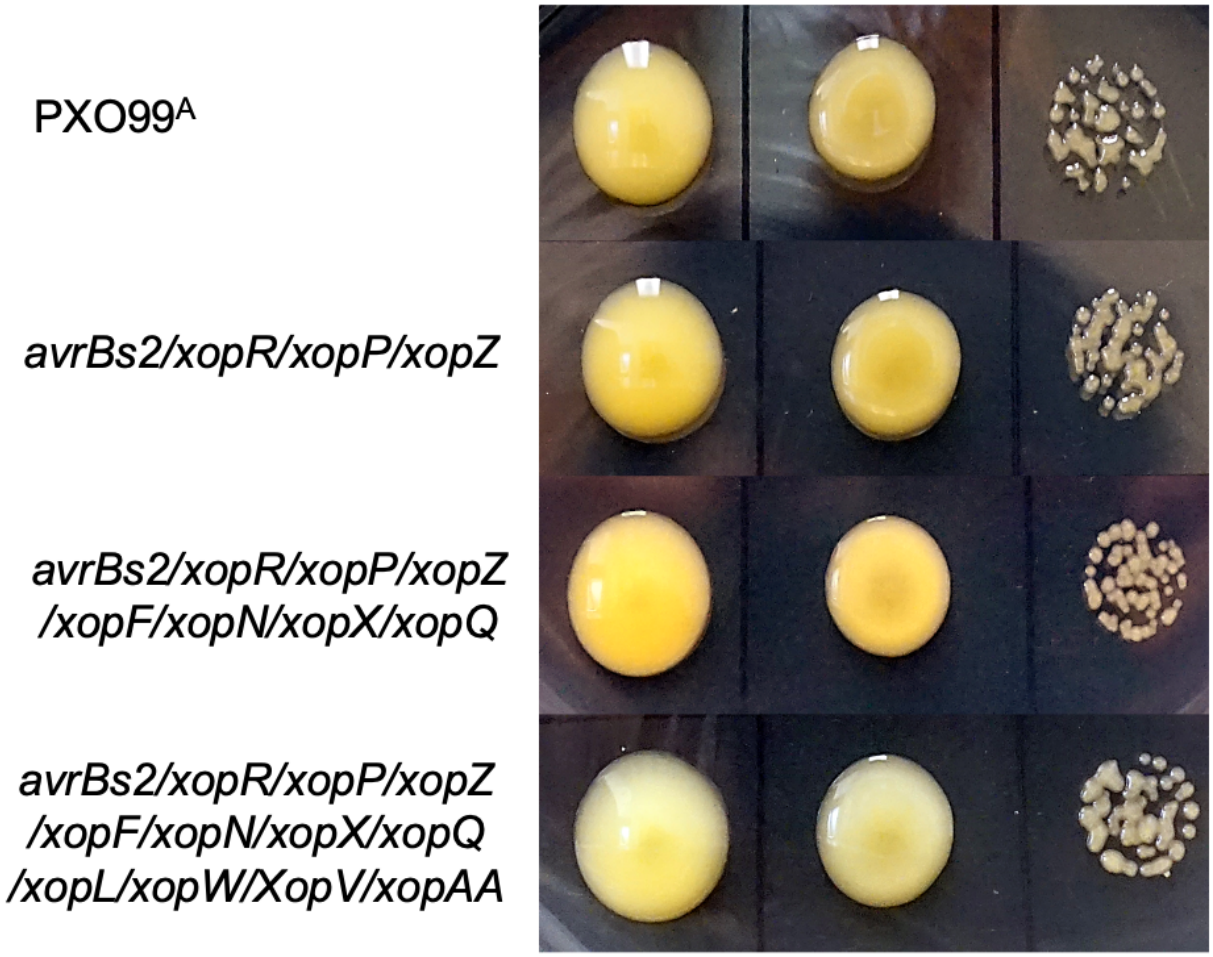

Supplementary Figure 19. Phenotypes of PXO99^{A} and derived type III effector gene mutants.

Colony growth of PXO99^{A}, quadruple (*avrBs2/xopR/xopP/xopZ*), and octuple (*avrBs2/xopR/xopP/xopZ/xopF/xopN/xopX/xopQ*) mutants in TSA medium. An aliquot of 5ul of bacterial culture at OD_{600}=0.6 was serially diluted ten times and dropped on the medium. Photos were taken 24h after plating.

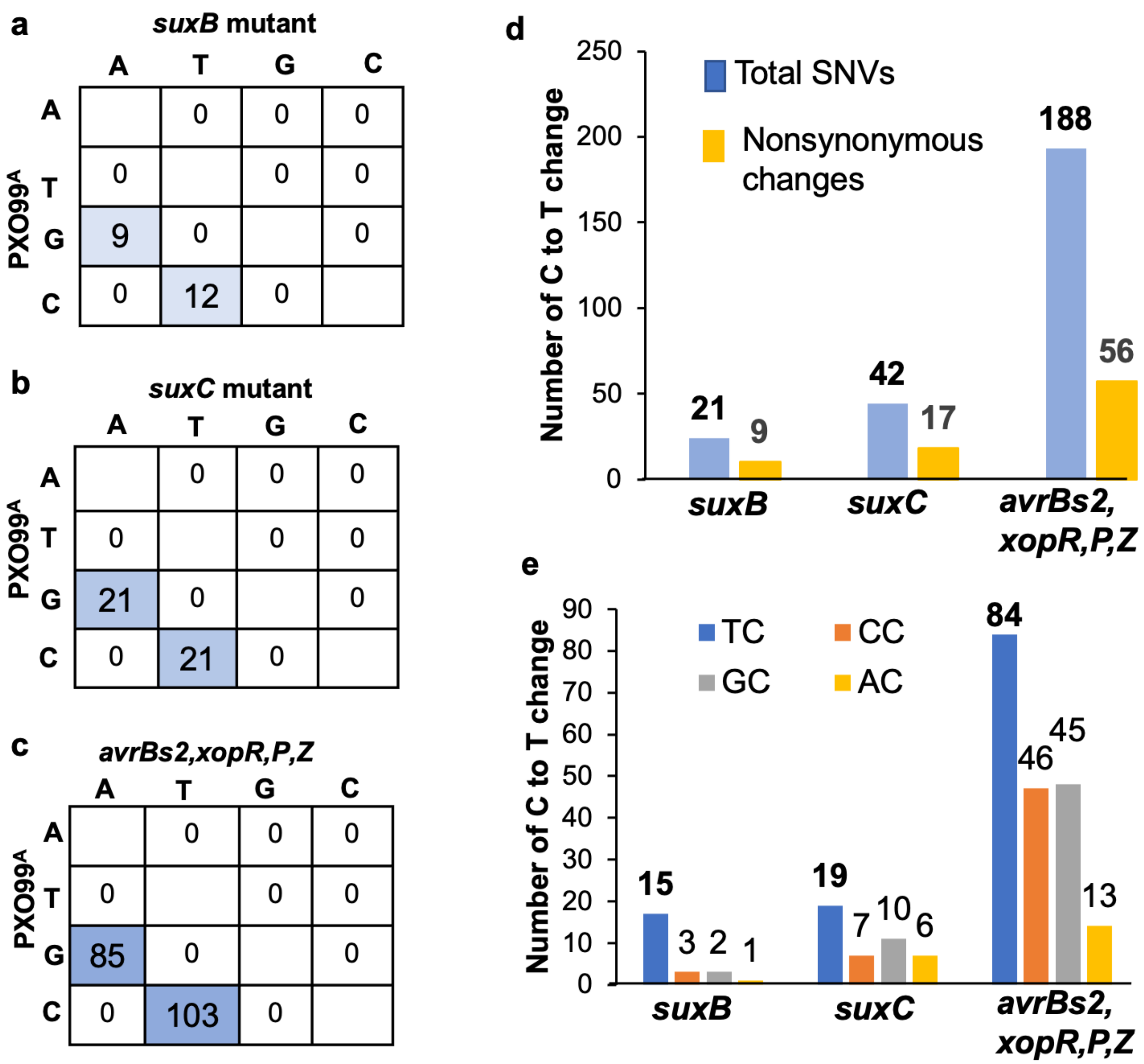

Supplementary Figure 20. Off-target analysis of base-edited genomes by whole-genome sequencing.

a, b, c Total number of SNVs detected in *suxB* mutant (**a**), *suxC* mutant (**b**) and mutant with four genes edited (**c**), respectively, compared to their progenitor PXO99^{A}.
d Total SNVs and nonsynonymous SNVs leading to changes in amino acids.
e Context effect of two nucleotides in four combinations (TC, CC, GC and AC) on C/G to T/A conversion in the edited genomes. The numbers indicate the total SNVs.

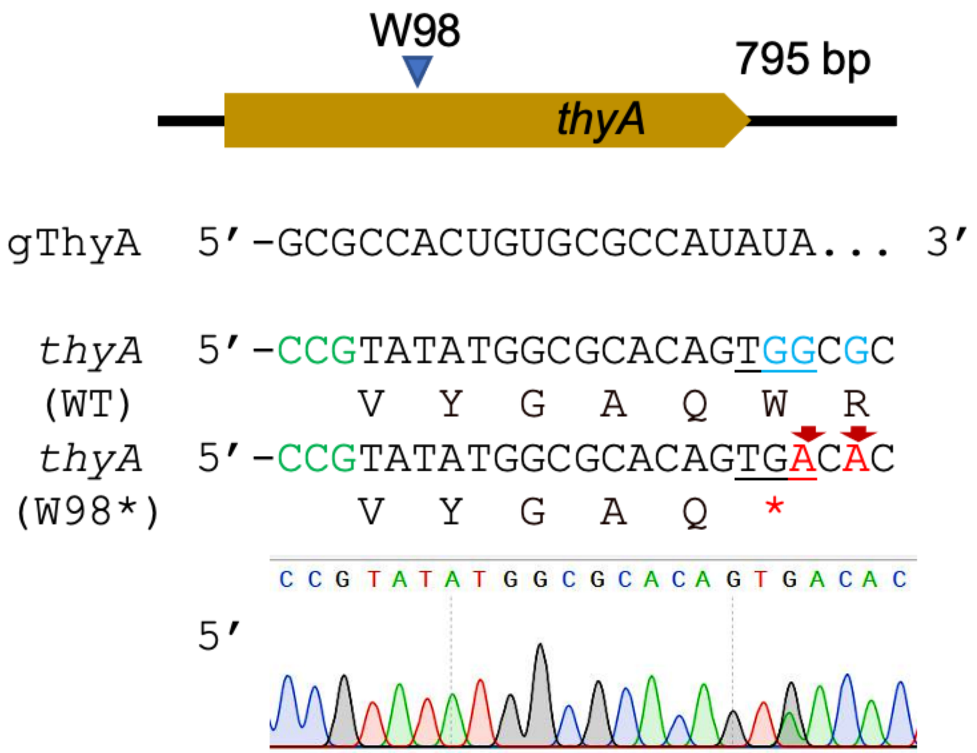

Supplementary Figure 21. Base editing in *A. tumefaciens* (LBA4404).
Outcome of based editing in *thyA* after eviction of gThyA. Guide RNAs and target sequences with PAM (in green) are also shown. Converted A (in red) from G (in blue) is indicated. Single letters for amino acids are used with asterisk for stop codon. An example of the chromatogram for *thyA* to show the G to A conversion is provided for gThyA.

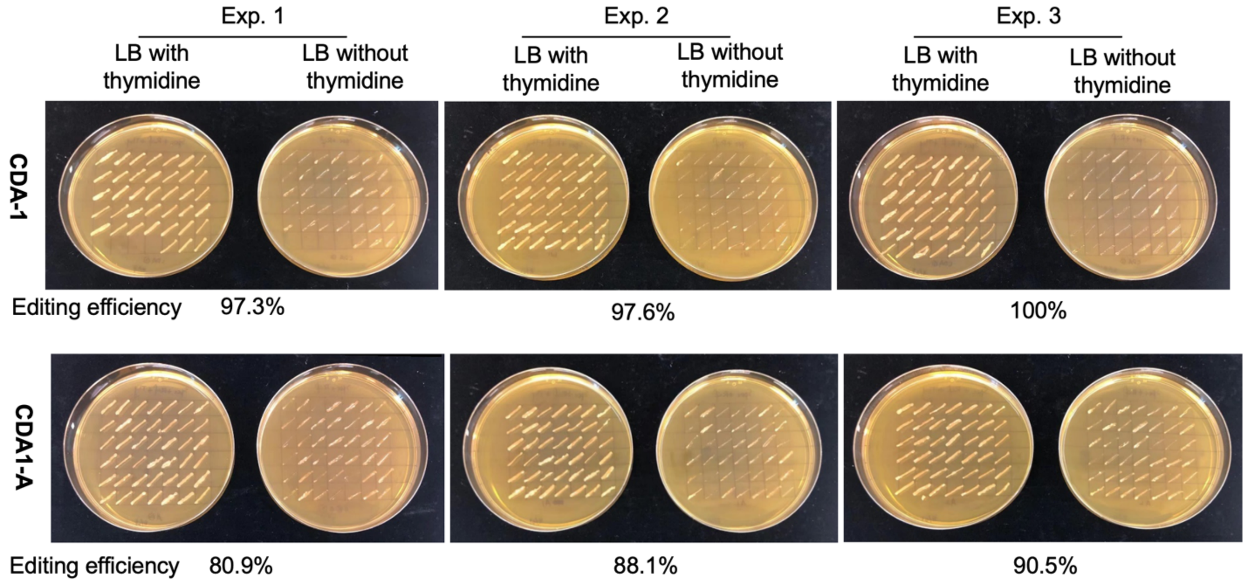

Supplementary Figure 22. On-target editing efficiency of CDA1 and engineered CDA1-A in *A. tumefaciens*.

LBA4404 colonies carrying gThyA and dCas9-CDA1 or dCas9-CDA1-A were streaked on duplicate plates supplemented with thymidine or lack thereof. Individual bacterial streaks with reduced or severely reduced growth were scored as edited events. The editing efficiency is presented as percentage of edited events of bacterial streaks grown on LB with thymidine.

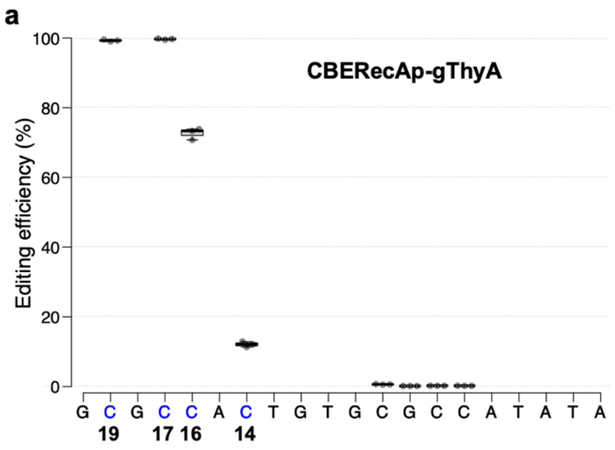

Supplementary Figure 23. Efficiency of base editing by CDA1 and engineered CDA1-A derived base editors.

Quantification of the C to T editing before eviction of gRNA as indicated (**a**, CDA1; **b**, CDA1-A). The percentage of C to T conversion is based on deep sequencing of 15 individual transformants in three pools. Edited C's in the protospacers are indicated by with the numbers for the positions relative to the PAM.

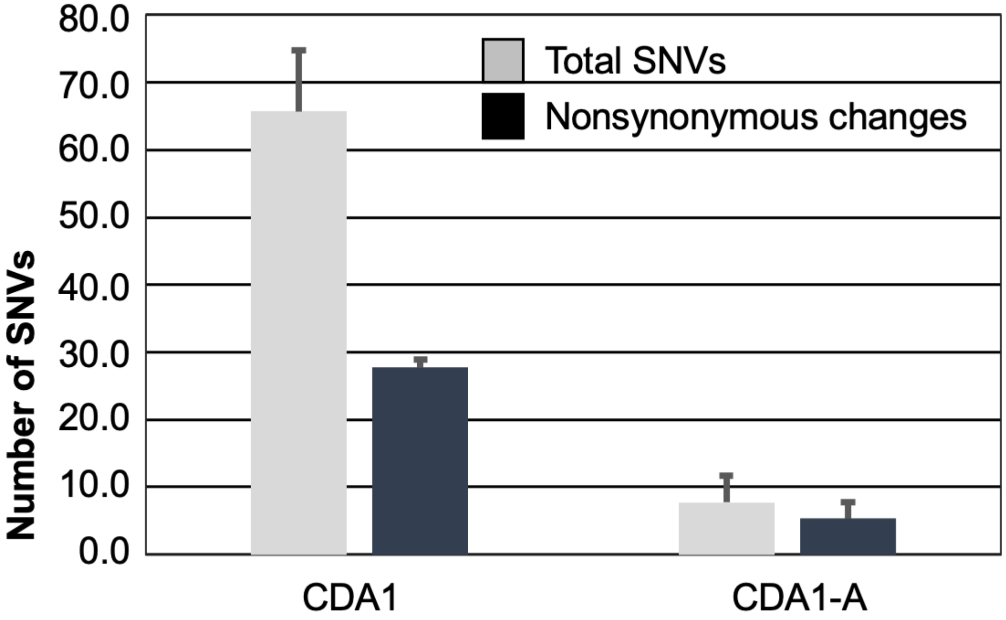

Supplementary Figure 24. Total SNVs and nonsynonymous SNVs in LBA4404.
Total SNVs and the nonsynonymous ones in *Agrobacterium* LBA4404 caused by CDA1 and engineered CDA1-A derived base editors.

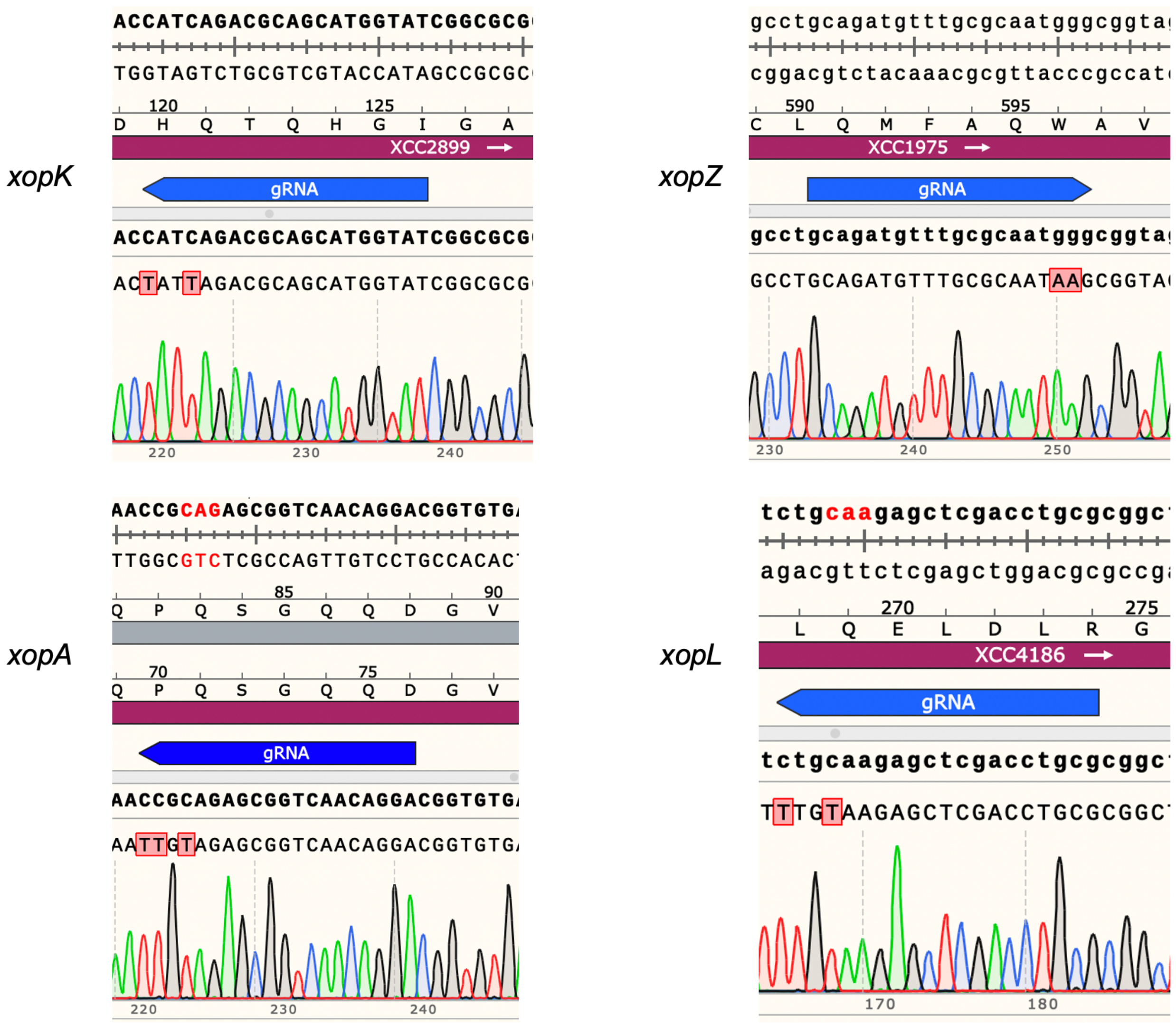

Supplementary Figure 25. Efficiency of multiplex base editing by the engineered CDA1-A derived base editor, CBE_{RecAp}-A.

Four *xop* (Xanthomonas outer protein) genes (*xopK*, *xopZ*, *xopA* and *xopL*) from *X. campestris* pv. *campestris* (*Xcc*) were successfully base edited using the CBE_{RecAp}-A. The partial image of Sanger sequencing chromatogram aligned to the reference gene sequence is shown. The blue bar indicates the gRNA used for base editing; the highlighted sequences are edited bases from the selected transformant after evicting of base editor. The single strain has all four genes edited.

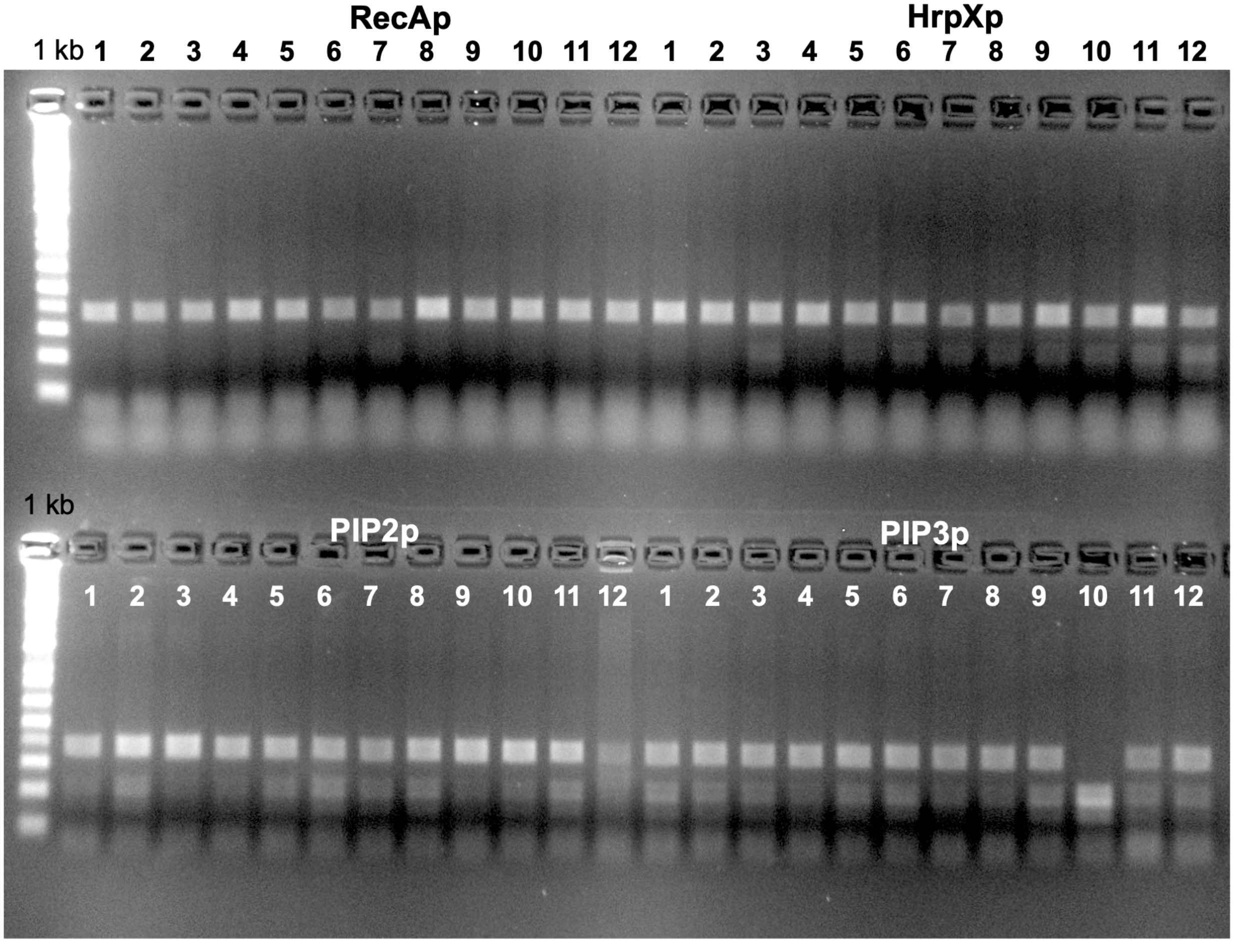

Supplementary Figure 26. Uncropped gel image for Supplementary Figure 4a.
Twelve samples from each of CBE driven by different promoters were loaded as indicated above the lanes.

Supporting Tables

Supplementary Table 1. Bacterial strains and plasmids used in this study.

Name	Features	Source (ref.)
**Bacterial strain**		
Escherichia coli EPI300	tonA^{-}/inducible *trf*A^{-}/*ori*V	Invitrogen
E. coli DB3.1	F-φ80(lacZ)ΔM15ΔlacX74hsdR (rk^{-}, mk^{+}) ΔrecA1398endA1tonA Str^{R}, *gyrA*462 *endA*1 Δ(*sr*1-*rec*A) *mcrB mrr hsdS*20 *glnV*44 *ara*14 *galK*2 *lac*Y1 *proA*2 *rps*L20 *xyl*5 *leuB*6 *mtl*1, resistant to ccdB	1
X. oryze pv *oryzae* PXO99^{A}	Philippine race 6, azacytidine resistant clone of PXO99	2
PXO99^{A}-suxB (Q92*)		This work
PXO99^{A}-suxC(Q145*)		This work
PXO99^{A}-xopF(Q38*)/xopN(Q203*)		This work
PXO99^{A}-avrBs2(Q29*)/xopP(W53*)/xopR(W158*)/W814*)		This work
P. syringae pv *tomato* DC3000	Wild type Rif^{R}	3
DC3000-avrPto(Q42*)	CRISPR single mutant	This work
DC3000-avrPto(Q86*)	CRISPR single mutant	This work
DC3000-hopK1(Q61*)	CRISPR single mutant	This work
DC3000-hopK1(Q155*)	CRISPR single mutant	This work
DC3000-avrPto(Q42*)/hopK1(Q61*)	CRISPR double mutant	This work
E. amylovora Ea9	Wild type	Yang lab
Ea9-dspE/A(Q239*)	CRISPR single mutant	This work
Ea9-dspE/A(W433*)	CRISPR single mutant	This work
Agrobacteria tumefaciens LBA4404	Wild type	Yang lab
LBA4404-thyA(W98*)	CRISPR single mutant	This work
**Plasmid**		
pEN-L4-PvirB-dCas9-UL-T3-R1	Multiplex Gateway entry clone, Kan^{R}	4
pEN-L1-PJ23119-BsaI-PglpT-sfGFP-TrrfB-BsaI-Scaf-L2	Shuttle cloning vector for gRNA, Kan^{R}	4

pEN-R2-SacB-L3	Multiplex Gateway entry clone, Kan^R	4
pPm43GW	Destination vector, Spe^R	4
pHM-Gib	ccdB and high-copy-number ColE1/pMB1/pBR322/pUC origin of replication	5
pEN-L4-RecAp-dCas9-CDA1-T1	MultiSite Gateway entry clone, Kan^R	This work
pEN-L4-HrpXp-dCas9-CDA1-T1	MultiSite Gateway entry clone, Kan^R	This work
pEN-L4-PIP2p-dCas9-CDA1-T1	MultiSite Gateway entry clone, Kan^R	This work
pEN-L4-PIP3p-dCas9-CDA1-T1	MultiSite Gateway entry clone, Kan^R	This work
pHM1-attR3R4	Destination vector, Kan^R	This work
pTL-Bag1	Shuttle cloning vector for gRNA, Kan^R	This work
pTL-Bag2	Shuttle cloning vector for gRNA, Kan^R	This work
pTL-Bag2T	Shuttle cloning vector for gRNA, Kan^R	This work
pTL-Bag3	Shuttle cloning vector for gRNA, Kan^R	This work
pTL-Bag4	Shuttle cloning vector for gRNA, Kan^R	This work
pEN-L4-RecAp-dCas9-CDA1-A(S30A)	Multiplex Gateway entry clone, Kan^R	This work
pEN-L4-RecAp-dCas9-CDA1-AA(S30A+H31A)	Multiplex Gateway entry clone, Kan^R	This work
pEN-L4-RecAp-dCas9-CDA1-YE(W94Y+R133E)	Multiplex Gateway entry clone, Kan^R	This work
pEN-L4-RecAp-dCas9-CDA1-YEE(W94E+R133E+W139E)	Multiplex Gateway entry clone, Kan^R	This work

Note:
For the bacterial strains, the features, such as antibiotic resistance, plasmid harbored, and source, are detailed. For plasmids, antibiotic resistances, the functional roles, and the source are provided. Km^R, kanamycin resistance; Rif^R, rifampicin resistance; Tet^R, tetracycline resistance; Sp^R, spectinomycin resistance; dCas, dead Cas9; CDA1, cytidine deaminase.

Supplementary Table 2. gBlock sequences used in this study.

gBlock	Sequences (5´-3´)
BagRNA1	TGTTACATTGCACAAGATAAAAATATATCATCATGCCTCCTCTAGAATG*GGTCTC*T**TAGC**G*GAGACG*AAGCTTGAAGACCGTCGACCTGCAGACTGGCTGTGTATAAGGGAGCCTGACATTTATATTCCCC*CGTCTC*G**GTTT**TAGAGCTAGAAATAGCAAGTTAAAATAAGGCTAGTCCGTTATCAACTTGAAAAAGTGGCACCGAGTCGGTGCTTTTTTT**CTGA**G*GAGACC*ATGCTCGAGCCACCCATGACCAAAATCCCTTAACGTG
BagRNA2	TGTTACATTGCACAAGATAAAAATATATCATCATGCCTCCTCTAGAatg*GGTCTC*T**CTGA**TTGACAGCTAGCTCAGTCCTAGGTATAATGC**TAGC**G*GAGACG*AAGCTTGaAGACCGTCGACCTGCAGACTGGCTGTGTATaAgGGAGCCTGACATTTATATTCCCC*CGTCTC*g**GTTT**TAGAGCTAGAAATAGCAAGTTAAAATAAGGCTAGTCCGTTATCAACTTGAAAAAGTGGCACCGAGTCGGTGCTTTTTTT**AAGA**GGAGACCATGCTCGAGCCACCCATGACCAAAATCCCTTAACGTG
BagRNA2T	TGTTACATTGCACAAGATAAAAATATATCATCATGCCTCCTCTAGAATG*GGTCTC*T**GACT**TTGACAGCTAGCTCAGTCCTAGGTATAATGC**TAGC**G*GAGACG*AAGCTTGaAGACCGTCGACCTGCAGACTGGCTGTGTATaAgGGAGCCTGACATTTATATTCCCC*CGTCTC*G**GTTT**GGAGACCATGCTCGAGCCACCCATGACCAAAATCCCTTAACGTG
BagRNA3	TGTTACATTGCACAAGATAAAAATATATCATCATGCCTCCTCTAGAATG*GGTCTC*T**AAGA**TTGACAGCTAGCTCAGTCCTAGGTATAATGC**TAGC**G*GAGACG*AAGCTTGaAGACCGTCGACCTGCAGACTGGCTGTGTATaAgGGAGCCTGACATTTATATTCCCC*CGTCTC*g**GTTT**TAGAGCTAGAAATAGCAAGTTAAAATAAGGCTAGTCCGTTATCAACTTGAAAAAGTGGCACCGAGTCGGTGCTTTTTTT**GACT**G*GAGACC*ATGCTCGAGCCACCCATGACCAAAATCCCTTAACGTG
BagRNA4	TGTTACATTGCACAAGATAAAAATATATCATCATGCCTCCTCTAGAATG*GGTCTC*T**GACT**TTGACAGCTAGCTCAGTCCTAGGTATAATGC**TAGC**G*GAGACG*AAGCTTGaAGACCGTCGACCTGCAGACTGGCTGTGTATAAGGGAGCCTGACATTTATATTCCCC*CGTCTC*G**GTTT**G*GAGACC*ATGCTCGAGCCACCCATGACCAAAATCCCTTAACGTG
RecAp	ACGTTGTAAAACGACGGCCAGTCTTAAGTAATGAgctctgtaccgatggtcgtgctggctttttcccgagcaggcaccgcagatttcccccctgccgcgggacATGGCGtctccctaatatgagtgtgtcgcctgcagtctcagcctgtgcgatacaccgccgctaCATTAGccccataccgaaatcactgacGAGGAtcacccag**ATG**GATAAGAAATACTCAATAGGCTTAGCTATCGGCACAAATAGCGTCGGATGGGCGGTGATCACTGATGAATATAAGGTTCCGTCTAAAAAGTTCAAGGTTCTGGGAAATACAGACCGCCACAGTATCAAAAAAAATCTTATAGGGGCTCTTTTATTTGACAGTGGAGAGACAGCGGAAGCGACTCGTCTCAAACGGACAGCTCGTAGAAGGTATACACGTCGGAAGAATCGTATT
HrpXp	ACGTTGTAAAACGACGGCCAGTCTTAAGTAATGAgaacagagtgaggtttttttgatttttttcagtctcttcagttgacaaattccattggatccgctgcatacaatcgtgtgcgccagcgagctcggcgattgttgtcttttgctccgcccccccaagagagagaccggc**ATG**GATAAGAAATACTCAATAGGCTTAGCTATCGGCACAAATAGCGTCGGATGGGCGGTGATCACTGATGAATATAAGGTTCCGTCTAAAAAGTTCAAGGTTCTGGGAAATACAGACCGCCACAGTATCAAAAAAAATCTTATAGGGGCTCTTTTATTTGACAGTGGAGAGACAGCGGAAGCGACTCGTCTCAAACGGACAGCTCGTAGAAGGTATACACGTCGGAAGAATCGTATT
PIP1p	ACGTTGTAAAACGACGGCCAGTCTTAAGTAATGAagagcttatccgctccccaccgcgactgcgcgggagcgggctcctgccgagtgtgatcaggttgggcaggtctgccagtgtcatgcatcgcgcttccatccagcttcgcaagttctgcagcttttttcggctgggtgggagcgtttttggccagggtctgtcaatttagcggcgtttgcatgaatgaggtgtcagccc**ATG**GATAAGAAATACTCAATAGGCTTAGCTATCGGCACAAATAGCGTCGGATGGGCGGTGATCACTGATGAATATAAGGTTCCGTCTAAAAAGTTCAAGGTTCTGGGAAATACAGACCGCCACAGTATCAAAAAAAATCTTATAGGGGCTCTTTTATTTGACAGTGGAGAGACAGCGGAAGCGACTCGTCTCAAACGGACAGCTCGTAGAAGGTATACACGTCGGAAGAATCGTATT
PIP2p	ACGTTGTAAAACGACGGCCAGTCTTAAGTAATGAcgcgacactcgacagtactgcaactgcgcgatatttggcttttccggcgtttccgatggcgcttagcgtccgttcgccaggagacgcgtaactgcggctacgaacaaaattcccgacttgcaagtcgttagcatccacggtactacacctgacggcatgcgattggcaatccatccgcaatccttcccgatgttaaagaaaaatctcttgagaagtcca**ATG**GATAAGAAATACTCAATAGGCTTAGCTATCGGCACAAATAGCGTCGGATGGGCGGTGATCACTGATGAATATAAGGTTCCGTCTAAAAAGTTCAAGGTTCTGGGAAATACAGACCGCCACAGTATCAAAAAAAATCTTATAGGGGCTCTTTTATTTGACAGTGGAGAGACAGCGGAAGCGACTCGTCTCAAACGGACAGCTCGTAGAAGGTATACACGTCGGAAGAATCGTATT

PIP3p	ACGTTGTAAAACGACGGCCAGTCTTAAGTAATGAgggcgtggtcgtagtgggtgccctggtcggc<br>aggctgggagttgccgaacgcttgggtctgcgcggctggttggggctggcactggcggcccccgc<br>gatcgctgcgatcgcatgggggatgcgtctggcttgacgctcgcgctttcggactgcgcgacgcc<br>ggttcgccacccgtcttcggcaccgcaatgggatcgctacgatggatggacagtccgaaaggagt<br>aagtg**ATG**GATAAGAAATACTCAATAGGCTTAGCTATCGGCACAAATAGCGTCGGATGGGCGGTG<br>ATCACTGATGAATATAAGGTTCCGTCTAAAAAGTTCAAGGTTCTGGGAAATACAGACCGCCACAG<br>TATCAAAAAAAATCTTATAGGGGCTCTTTTATTTGACAGTGGAGAGACAGCGGAAGCGACTCGTC<br>TCAAACGGACAGCTCGTAGAAGGTATACACGTCGGAAGAATCGTATT
*att*L4	TGATGCCTGGCAGTTCCCTACTCTCGCGTTAACAGATCTTACCATGGAGCTCCAAATAATGATTT<br>TATTTTGACTGATAGTGACCTGTTCGTTGCAACAAATTGATAAGCAATGCTTTTTTATAATGCCA<br>ACTTTGTATAGAAAAGTTGCTACCTCGAGCACTAGTAACGCTAGCATGGATGTTTTCCCAGTC
CDA1-YE	ATCCTGAGAATCCGGGATAAGCCTGAGGAGATGACCGACGCTGAGTACGTGAGAATCCATGAGAA<br>GTTGGACATCTACACGTTTAAGAAACAGTTTTTCAACAACAAAAAATCCGTGTCGCATAGATGCT<br>ACGTTCTCTTTGAATTAAAACGACGGGGTGAACGTAGAGCGTGTTTTTGGGGCTATGCTGTGAAT<br>AAACCACAGAGCGGGACAGAACGTGGCATTCACGCCGAAATCTTTAGCATTAGAAAAGTCGAAGA<br>ATACCTGCGCGACAACCCCGGACAATTCACGATAAATTGGTACTCATCCTacAGTCCTTGTGCAG<br>ATTGCGCTGAAAAGATCTTAGAATGGTATAACCAGGAGCTGCGGGGGAACGGCCACACTTTGAAA<br>ATCTGGGCTTGCAAACTCTATTACGAGAAAAATGCGgaGAATCAAATTGGGCTGTGGAATCTCAG<br>AGATAACGGGGTTGGGTTGAATGTAATGGTAAGTGAACACTACCAATGTTGCAGGAAAATATTCA<br>TCCAATCGTCGCACAATCAATTGAATGAGAATAGATGGCTTGAGAAGACTTTGAAGCGAGCTGAA<br>AAACGACGGAGCGAGTTGTCCATTATGATTCAGGTAAAAATACTCCACACCACTAAGAGTCCTGC<br>TGTTTCTAGAATGACCAACCTTTCCGACATCATAGAGAAGGAAACAGGCAAACAGTTGGTCATCC<br>AAGAGTCGATACTCATGCTTCCTGAAGAAGTTGAGGAGGTCATTGGGAATAAGCCGGAAAGTGAC<br>ATTCTCGTACACACTGCGTATGATGAGAGCACCGATGAGAACGTGATGCTGCTCACGTCAGATGC<br>CCCAGAGTACAAACCCTGGGCTCTGGTGATTCAGGACTCTAATGGAGAGAACAAGATCAAGATGC<br>TAGGACTGGTTGCATAATCAGAATAAGCAAACCCCTTGGGTCAGTATGCAAGTTTGTACAAAAAA<br>GTTGAACGAGAAACGTAAAATG
CDA1-YEE	ATCCTGAGAATCCGGGATAAGCCTGAGGAGATGACCGACGCTGAGTACGTGAGAATCCATGAGAA<br>GTTGGACATCTACACGTTTAAGAAACAGTTTTTCAACAACAAAAAATCCGTGTCGCATAGATGCT<br>ACGTTCTCTTTGAATTAAAACGACGGGGTGAACGTAGAGCGTGTTTTTGGGGCTATGCTGTGAAT<br>AAACCACAGAGCGGGACAGAACGTGGCATTCACGCCGAAATCTTTAGCATTAGAAAAGTCGAAGA<br>ATACCTGCGCGACAACCCCGGACAATTCACGATAAATTGGTACTCATCCTacAGTCCTTGTGCAG<br>ATTGCGCTGAAAAGATCTTAGAATGGTATAACCAGGAGCTGCGGGGGAACGGCCACACTTTGAAA<br>ATCTGGGCTTGCAAACTCTATTACGAGAAAAATGCGgaGAATCAAATTGGGCTGgaGAATCTCAG<br>AGATAACGGGGTTGGGTTGAATGTAATGGTAAGTGAACACTACCAATGTTGCAGGAAAATATTCA<br>TCCAATCGTCGCACAATCAATTGAATGAGAATAGATGGCTTGAGAAGACTTTGAAGCGAGCTGAA<br>AAACGACGGAGCGAGTTGTCCATTATGATTCAGGTAAAAATACTCCACACCACTAAGAGTCCTGC<br>TGTTTCTAGAATGACCAACCTTTCCGACATCATAGAGAAGGAAACAGGCAAACAGTTGGTCATCC<br>AAGAGTCGATACTCATGCTTCCTGAAGAAGTTGAGGAGGTCATTGGGAATAAGCCGGAAAGTGAC<br>ATTCTCGTACACACTGCGTATGATGAGAGCACCGATGAGAACGTGATGCTGCTCACGTCAGATGC<br>CCCAGAGTACAAACCCTGGGCTCTGGTGATTCAGGACTCTAATGGAGAGAACAAGATCAAGATGC<br>TAGGACTGGTTGCATAATCAGAATAAGCAAACCCCTTGGGTCAGTATGCAAGTTTGTACAAAAAA<br>GTTGAACGAGAAACGTAAAATG
CDA1-A	ATCCTGAGAATCCGGGATAAGCCTGAGGAGATGACCGACGCTGAGTACGTGAGAATCCATGAGAA<br>GTTGGACATCTACACGTTTAAGAAACAGTTTTTCAACAACAAAAAATCCGTGgctCATAGATGCT<br>ACGTTCTCTTTGAATTAAAACGACGGGGTGAACGTAGAGCGTGTTTTTGGGGCTATGCTGTGAAT<br>AAACCACAGAGCGGGACAGAACGTGGCATTCACGCCGAAATCTTTAGCATTAGAAAAGTCGAAGA<br>ATACCTGCGCGACAACCCCGGACAATTCACGATAAATTGGTACTCATCCTGGAGTCCTTGTGCAG<br>ATTGCGCTGAAAAGATCTTAGAATGGTATAACCAGGAGCTGCGGGGGAACGGCCACACTTTGAAA<br>ATCTGGGCTTGCAAACTCTATTACGAGAAAAATGCGAGGAATCAAATTGGGCTGTGGAATCTCAG<br>AGATAACGGGGTTGGGTTGAATGTAATGGTAAGTGAACACTACCAATGTTGCAGGAAAATATTCA<br>TCCAATCGTCGCACAATCAATTGAATGAGAATAGATGGCTTGAGAAGACTTTGAAGCGAGCTGAA<br>AAACGACGGAGCGAGTTGTCCATTATGATTCAGGTAAAAATACTCCACACCACTAAGAGTCCTGC<br>TGTTTCTAGAATGACCAACCTTTCCGACATCATAGAGAAGGAAACAGGCAAACAGTTGGTCATCC<br>AAGAGTCGATACTCATGCTTCCTGAAGAAGTTGAGGAGGTCATTGGGAATAAGCCGGAAAGTGAC<br>ATTCTCGTACACACTGCGTATGATGAGAGCACCGATGAGAACGTGATGCTGCTCACGTCAGATGC

	CCCAGAGTACAAACCCTGGGCTCTGGTGATTCAGGACTCTAATGGAGAGAACAAGATCAAGATGC<br>TAGGACTGGTTGCATAATCAGAATAAGCAAACCCCTTGGGTCAGTATGCAAGTTTGTACAAAAAA<br>GTTGAACGAGAAACGTAAAATG
CDA1-AA	ATCCTGAGAATCCGGGATAAGCCTGAGGAGATGACCGACGCTGAGTACGTGAGAATCCATGAGAA<br>GTTGGACATCTACACGTTTAAGAAACAGTTTTTCAACAACAAAAAATCCGTGgctgcTAGATGCT<br>ACGTTCTCTTTGAATTAAAACGACGGGGTGAACGTAGAGCGTGTTTTTGGGGCTATGCTGTGAAT<br>AAACCACAGAGCGGGACAGAACGTGGCATTCACGCCGAAATCTTTAGCATTAGAAAAGTCGAAGA<br>ATACCTGCGCGACAACCCCGGACAATTCACGATAAATTGGTACTCATCCTGGAGTCCTTGTGCAG<br>ATTGCGCTGAAAAGATCTTAGAATGGTATAACCAGGAGCTGCGGGGGAACGGCCACACTTTGAAA<br>ATCTGGGCTTGCAAACTCTATTACGAGAAAAATGCGAGGAATCAAATTGGGCTGTGGAATCTCAG<br>AGATAACGGGGTTGGGTTGAATGTAATGGTAAGTGAACACTACCAATGTTGCAGGAAAATATTCA<br>TCCAATCGTCGCACAATCAATTGAATGAGAATAGATGGCTTGAGAAGACTTTGAAGCGAGCTGAA<br>AAACGACGGAGCGAGTTGTCCATTATGATTCAGGTAAAAATACTCCACACCACTAAGAGTCCTGC<br>TGTTTCTAGAATGACCAACCTTTCCGACATCATAGAGAAGGAAACAGGCAAACAGTTGGTCATCC<br>AAGAGTCGATACTCATGCTTCCTGAAGAAGTTGAGGAGGTCATTGGGAATAAGCCGGAAAGTGAC<br>ATTCTCGTACACACTGCGTATGATGAGAGCACCGATGAGAACGTGATGCTGCTCACGTCAGATGC<br>CCCAGAGTACAAACCCTGGGCTCTGGTGATTCAGGACTCTAATGGAGAGAACAAGATCAAGATGC<br>TAGGACTGGTTGCATAATCAGAATAAGCAAACCCCTTGGGTCAGTATGCAAGTTTGTACAAAAAA<br>GTTGAACGAGAAACGTAAAATG

Note:
The 5′overhangs for Golden Gate assembly are in **bold** and highlighted in grey. *Xba* I and *Xho* I restriction sites are underlined and *italic*, *Bsa* I restriction sites are underlined, *italic,* and highlighted by in green. *BsmB*I restriction sites are underlined, *italic*, and highlighted by in blue. The overlapping sequences for Gibson assembly are highlighted by in yellow. The start codons for dCas9 under control of individual promoters are in red and bold; the individual promoter sequences are lower letters. CDA1-variation gBlocks were incorporated into pEN-L4-RecAp-dCas9-CDA-T1 at *Bsu36* I recognition site (sequence in green) and *BsrG* I recognition site (sequence in blue) through Gibson Assembly.

Supplementary Table 3. Oligonucleotides used in this study.

Name	Sequences (5´-3´)	Usage
gSuxB-F	**TAGC**GACGCAGTGGCTGGGAGAAC	gRNA for *suxB* in PXO99^{A}
gSuxB-R	**AAAC**GTTCTCCCAGCCACTGCGTC	
SuxB-F	ATCAGCCTGCGTTACGACTT	Genotyping *suxB*
SuxB-R	GTGAGTGCGATCAAATCGTC	
gSuxC-F	**TAGC**GCTCACGCAGAGTGCGTTCAC	gRNA for *suxC* in PXO99^{A}
gSuxC-R	**AAAC**GTGAACGCACTCTGCGTGAGC	
SuxC-F	CATGGTCGGTGGCATCTC	Genotyping *suxC*
SuxC-R	GCAACATGAGCCCGATCTAC	
gHopK1-F1	**TAGC**CCAGCGACTGCGCCAATTGA	gRNAs for *hopK1* in DC3000
gHopK1-R1	**AAAC**TCAATTGGCGCAGTCGCTGG	
gHopK1-F2	**TAGC**GATCAGGACCGCGCGCCCTTC	
gHopK1-R2	**AAAC**GAAGGGCGCGCGGTCCTGATC	
HopK1-F1	CCGACAATTCCCCTCTAGTT	Genotyping *hopK1*
HopK1-R1	GCTGCCTCTAGGCACAAACT	
gAvrPto-F1	**TAGC**CATCAACTTGCGGAGTCTGC	gRNAs for *avrPto* in DC3000
gAvrPto-R1	**AAAC**GCAGACTCCGCAAGTTGATG	
gAvrPto-F2	**TAGC**CATGCAGCATAGGTACATGA	
gAvrPto-R2	**AAAC**TCATGTACCTATGCTGCATG	
AvrPto-F1	ATGGCCCATCAGGTGAACT	Genotyping *avrPto*
AvrPto-R1	CATTGCGTACTGCAGAGCTT	
gDspE/A-F1	**TAGC**TCCAGCAACTGCATCAACAG	gRNAs for *dspE/A* in *Erwinia* E9
gDspE/A-R1	**AAAC**CTGTTGATGCAGTTGCTGGA	
gDspE/A-F2	**TAGC**CTGCCAAATGCCGGTTAATA	
gDspE/A-R2	**AAAC**TATTAACCGGCATTTGGCAG	
DspE/A-F1	AAGGAAGAACCGGTTGGCTC	Genotyping *dspE/A*
DspE/A-R1	CAGCTGGCTGTGGGTATCTT	
gAvBs2-F	**TAGC**GACGCAGGTGCCGCACGTGC	gRNA for *avrBs2* in PXO99^{A}
gAvBs2-R	**AAAC**GCACGTGCGGCACCTGCGTC	
AvrBs2-F1	GTGTGAAACGCATCGAAACC	Genotyping *avrBs2*
AvrBs2-R1	CAGTGTCTGCACGGTGATCT	
gXopR-F	**TAGC**GTTGCCATCTCTCTTCGTTG	gRNA for *xopR* in PXO99^{A}
gXopR-R	**AAAC**CAACGAAGAGAGATGGCAAC	
XopR-F1	GTAAGGCATCCACACCCCAC	Genotyping *xopR*
XopR-R1	AGGGTCTTCACGGGTATCGA	
gXopP-F	**TAGC**GCCATGGCGCTCGATCCGC	gRNA for *xopP* in PXO99^{A}
gXopP-R	**AAAC**GCGGATCGAGCGCCATGGC	
XopP-F1	GATTTCGCTGCACGTCATCC	Genotyping *xopP*
XopP-R1	TGACGTTCTTTGCGTTGCTG	
gXopZ-F	**TAGC**ACCCAGGCGGACGCGATCGC	

gXopZ-R	**AAAC**GCGATCGCGTCCGCCTGGGT	gRNA for *XopZ* in PXO99[A]
XopZ-F1	GCGTCGCCACATTCAAATGA	Genotyping *xopZ*
XopZ-R1	TATCGCGCCATTCCAATCGT	
gXopF-F	**TAGC**GCACAGACTGAACCGCTTTG	gRNA for *xopF* in PXO99[A]
gXopF-R	**AAAC**CAAAGCGGTTCAGTCTGTGC	
XopF-F1	GCAGGAGGTGGCATCATGAA	Genotyping *xopF*
XopF-R1	AAGGCAGTGATGCGTTCCTG	
gXopN-F	**TAGC**GCATCAGCCCTGGGCAGGCGC	gRNA for *xopN* in PXO99[A]
gXopN-R	**AAAC**GCGCCTGCCCAGGGCTGATGC	
XopN-F1	GCAACACATGCGGATCTGTT	Genotyping *xopN*
XopN-R1	CTTGCAGCACTTGATGAGCC	
gXopX-F	**TAGC**GAGCAAACGCACCTCGACAC	gRNA for *xopX* in PXO99[A]
gXopX-R	**AAAC**GTGTCGAGGTGCGTTTGCTC	
XopX-F	GCGACATCGGGCAGATAGAT	Genotyping *xopX*
XopX-R	GAGATCGACCATAGGCCGTG	
gXopQ-F	**TAGC**GAACCACGCCGTATCCAGGC	gRNA for *xopQ* in PXO99[A]
gXopQ-R	**AAAC**GCCTGGATACGGCGTGGTTC	
XopQ-F	TACAACAACGCCACCGACAT	Genotyping *xopQ*
XopQ-R	CTTTTTCCGGACGCACAAC	
gXopL-F1	**TAGC**GCCATTGCGACAAGACGTCG	gRNA for *xopL* in PXO99[A]
gXopL-R1	**AAAC**CGACGTCTTGTCGCAATGGC	
XopL-F1	GCAATTGCCACAACTCAGCA	Genotyping *xopL*
XopL-R1	GCTTGAGATGGATGCAGGGA	
gXopW-F1	**TAGC**GTTCAAGGTGAGTCTCGTC	gRNA for *xopW* in PXO99[A]
gXopW-R1	**AAAC**GACGAGACTCACCTTGAAC	
XopW-F1	CACATTTTTCCCATCCCGGC	Genotyping *xopW*
XopW-R1	CATCGCGCGCTAGATACTCA	
gXopV-F1	**TAGC**TGCCATGGGGTCTTGTTCCA	gRNA for *xopV* in PXO99[A]
gXopV-R1	**AAAC**TGGAACAAGACCCCATGGCA	
XopV-F1	CGATCTGCGCGACAAGAAAG	Genotyping of *xopV*
XopV-R1	ACGCTGCAACGGTATCTGTT	
gXopAA-F1	**TAGC**CAACAGCAGGCGATCCGACT	gRNA for *xopAA* in PXO99[A]
gXopAA-R1	**AAAC**AGTCGGATCGCCTGCTGTTG	
XopAA-F1	CGACGTTCCTGCTCAGTGAA	Genotyping of *xopAA*
XopAA-R1	CTGTGAGGGGCTGAAGAACA	
gThyA-F1	**TAGC**GCGCCACTGTGCGCCATATA	gRNA for thyA
gThyA-R1	**AAAC**TATATGGCGCACAGTGGCGC	

		In LBA4404
BagRNA2T-F	TGCCTCC*TCTAGA*ATG*GGTCTC*TCTGATTGACAG	Construct pTL-Bag2T
Seq-F	TGGCCCGTGTCTCAAAATCTCTG	Sequencing gRNA unit
Seq-R	ATCTTTTCTACGGGGTCTGACG	
MisuxB-F1	CTCTTTCCCTACACGACgctcttccgatctCTGGATGCCTTGCAGACGTT	PCR-ampli. for deep sequencing
MisuxB-R1	ctggagttcagacgtgtgctcttccgatctTAGCCGAGCATGTGCTGTTC	
MisuxC-F1	CTCTTTCCCTACACGACgctcttccgatctGGATGCGGCCAACAATGTG	PCR-ampli. for deep sequencing
MisuxC-R1	ctggagttcagacgtgtgctcttccgatctTCCTGACTCATGCCCATCCA	
MiavrptoF1	CTCTTTCCCTACACGACgctcttccgatctAACAACTCGGGTGACGAAGAT	PCR-ampli. for deep sequencing
MiavrptoR1	ctggagttcagacgtgtgctcttccgatctAGCATTCCCGGATTGATTCCT	
MiHopK-F1	CTCTTTCCCTACACGACgctcttccgatctCAACATCTATCGCCCAAGCG	PCR-ampli. for deep sequencing
MiHopK-R1	ctggagttcagacgtgtgctcttccgatctGAAGTGTCGTTGTTGGAGCG	
MiHopK-F2	CTCTTTCCCTACACGACgctcttccgatctAACTCCTTATTGGGCGGTGG	PCR-ampli. for deep sequencing
MiHopK-R2	ctggagttcagacgtgtgctcttccgatctCTCACGTTTGAGGCCAACAC	
MiDsp-F1	CTCTTTCCCTACACGACgctcttccgatctGGGTAGCCACCACGAAATCA	PCR-ampli. for deep sequencing
MiDsp-R1	ctggagttcagacgtgtgctcttccgatctGCTGAAACCTGGCGCTAATC	
MiDsp-F2	CTCTTTCCCTACACGACgctcttccgatctAGACGGTAAAAGCGGCAAGA	PCR-ampli. for deep sequencing
MiDsp-R2	ctggagttcagacgtgtgctcttccgatctTCATGCAGGCGGATTGACTC	
MiThyA-F1	CTCTTTCCCTACACGACgctcttccgatctTGGTTCCTGAAGGGCGATAC	PCR-ampli. for deep sequencing
MiThyA-R1	ctggagttcagacgtgtgctcttccgatctTCGATCAAAAGCGCGATCTG	
gXccK-F	tagcCATCAGACGCAGCATGGTAT	gRNA for *xopK*
gXccK-R	aaacATACCATGCTGCGTCTGATG	
gXccZ-F	tagcCCCATTGCGCAAACATCTGC	gRNA for *xopZ*
gXccZ-R	aaacGCAGATGTTTGCGCAATGGG	
gXccA-F	tagcCCGCAGAGCGGTCAACAGGA	gRNA for *xopA*
gXccA-R	aaacTCCTGTTGACCGCTCTGCGG	
gXccL-F	tagcCTGCAAGAGCTCGACCTGCG	gRNA for *xopL*
gXccL-R	aaacCGCAGGTCGAGCTCTTGCAG	
XccK-F1	CAAAGCTGGCGAAAATGCCA	Genotyping *xopK*
XccK-R1	ACTTCGCGGGTTTCCTTGTA	
XccZ-F1	CTCCCGGGAATGGTTGGAG	Genotyping *xopZ*
XccZ-R1	CAGTGAAGGTCAGGTCGGTC	
XccA-F1	GCGATGCATCCAGTTCAACC	Genotyping *xopA*
XccA-R1	CTGCACGCATGGTCAATCAG	
XccL-F1	CTCGCCACTTACCGAACTCA	Genotyping *xopL*
XccL-R1	GCTGCGAACAGAGCGTAAAC	

Note: Oligonucleotides are used to construct gRNA for base-editing applications, cloning of base-editing plasmids, and genotyping base-edited *Xanthomonas* (PXO99^{A}), *Psedoumonas* (DC3000), *Erwinia* (Ea9), *Agrobacterium* (LBA4404). Strains, confirmation of eviction of base-editing plasmids in *Xanthomonas* (PXO99^{A}), *Psedoumonas* (DC3000), *Erwinia* (Ea9), *Agrobacterium* (LBA4404). Strains and multiplexing applications. Golden Gate overhangs are in **bold**, and *Xba* I recognition site is underlined and *italic*, *Bsa* I recognition site is underlined, *italic,* and in green.

Supplementary References

1 P. Bernard, M. Couturier, Cell killing by the F plasmid CcdB protein involves poisoning of DNA-topoisomerase II complexes. *J Mol Biol* **226**, 735-745 (1992).

2 C. M. Hopkins, F. F. White, S. H. Choi, A. Guo, J. E. Leach, Identification of a family of avirulence genes from *Xanthomonas oryzae* pv. *oryzae. Mol Plant Microbe Interact* **5**, 451-459 (1992).

3 D. A. Cuppels, Generation and characterization of Tn*5* insertion mutations in *Pseudomonas syringae* pv. *tomato*. *Appl. Environ. Microbiol.* **51**, 323-327 (1986).

4 S. D. Rodrigues *et al.*, Efficient CRISPR-mediated base editing in *Agrobacterium* spp. *Pro Natl Acad Sci U S A* **118,** e2013338118 (2021).

5 C. Li *et al.*, An efficient method to clone TAL effector genes from genomes of *Xanthomonas oryzae* using Gibson assembly. *Mol Plant Pathol* **20**, 1453-1462 (2019).
